# Patient stratification based on urea cycle metabolism for exploration of combination immunotherapy in colon cancer

**DOI:** 10.1186/s12885-022-09958-7

**Published:** 2022-08-13

**Authors:** Zirui Zhao, Haohan Liu, Deliang Fang, Xingyu Zhou, Shaoji Zhao, Chaoyue Zhang, Jinning Ye, Jianbo Xu

**Affiliations:** Department of Gastrointestinal Surgery, First Affiliated Hospital of Sun Yat-Sen University, 510080 Guangzhou, Guangdong Province People’s Republic of China

**Keywords:** Urea cycle metabolism, Colon cancer, Hot tumors, Subdividing, Combination immunotherapy

## Abstract

**Background:**

Owing to the low ratio of patients benefitting from immunotherapy, patient stratification becomes necessary. An accurate patient stratification contributes to therapy for different tumor types. Therefore, this study aimed to subdivide colon cancer patients for improved combination immunotherapy.

**Methods:**

We characterized the patients based on urea cycle metabolism, performed a consensus clustering analysis and constructed a risk model in the cancer genome atlas cohort. Colon cancer patients were further categorized into two tags: clusters, and risk groups, for the exploration of combination immunotherapy. In addition to external validation in the Gene Expression Omnibus datasets, several images of immunohistochemistry were used for further validation.

**Results:**

Patient characterization based on urea cycle metabolism was related to immune infiltration. An analysis of consensus clustering and immune infiltration generated a cluster distribution and identified patients in cluster 1 with high immune infiltration levels as hot tumors for immunotherapy. A risk model of seven genes was constructed to subdivide the patients into low- and high-risk groups. Validation was performed using a cohort of 731 colon cancer patients. Patients in cluster 1 had a higher immunophenoscore (IPS) in immune checkpoint inhibitor therapy, and those other risk groups displayed varying sensitivities to potential combination immunotherapeutic agents. Finally, we subdivided the colon cancer patients into four groups to explore combination immunotherapy. Immunohistochemistry analysis showed that protein expression of two genes were upregulated while that of other two genes were downregulated or undetected in cancerous colon tissues.

**Conclusion:**

Using subdivision to combine chemotherapy with immunotherapy would not only change the dilemma of immunotherapy in not hot tumors, but also promote the proposition of more rational personalized therapy strategies in future.

**Supplementary Information:**

The online version contains supplementary material available at 10.1186/s12885-022-09958-7.

## Introduction

Obesity and unbalanced diets have significantly increased the incidence of colon cancer a lot [1, 2]. Colon cancer is the fourth most common cause of new cancer cases and cancer deaths globally (6.1% of 18.10 million new cancer cases and 5.8% of 9.6 million cancer deaths in 2018 worldwide) [3]. Approximately 25-50% of patients are diagnosed at an early stage of the disease but later develop metastasis [4]. Despite advances in colon cancer therapy, the survival rate is not high [5]. A new therapy strategy might change this situation. Immunotherapy, such as immune checkpoint inhibitor (ICI) therapy, has transformed the treatment landscape and offers significant clinical benefits for patients with multiple cancers. However, exploring a new method to stratify patients is essential because many biomarkers, such as tumor mutational burden (TMB), are less than satisfactory, and only one-third of patients benefit from ICI in most cancers [6–8].

Cancer-associated metabolic reprogramming has profound effects on gene expression and immune infiltration, leading to tumorigenesis [9]. With dysregulation of urea cycle (UC) metabolism, cancer cells maximize the use of nitrogen and carbon for tumor proliferation and growth. CAD is a protein composed of carbamoyl-phosphate synthetase 2 (CPS2), aspartate transcarbamylase, and dihydroorotase and can initiate pyrimidine synthesis. CAD utilizes an increasing number of UC substrates to increase the abundance of pyrimidines that regulate DNA expression and promote a specific identifiable mutagenic signature. This change contributes to the synthesis of hydrophobic neopeptides and improves anti-programmed cell death 1 immunotherapy, a type of ICI. In addition, dysregulation in the expression of UC genes can alter arginine levels and change immune cell activation, leading to different immunotherapy responses [6, 10, 11]. For example, small extracellular vesicles containing arginine can decrease T-cell activation in ovarian cancers; targeting arginine can enhance immunotherapy efficacy for leukemia [12, 13]. Furthermore, researchers are trying to combine ICI with pegylated arginine deiminase, an enzyme that can degrade arginine, to improve immunotherapy efficacy. Thus, in this study we attempted to stratify patients based on UC metabolism to improve combination immunotherapy for colon cancer.

Compared with the classical Tumor-Node-Metastasis (TNM) system, the observation that the type, density, and location of immune cells can predict survival more accurately in colon cancer, indicates that these parameters might serve as a better stratification index. Based on immune infiltration, we classified tumors into two types: hot tumors and not hot tumors. Hot tumors exhibit CD8^+^ T cell-infiltration (high immunoscore) and checkpoint activation. Owing to pre-existing immunity, hot tumors can induce immune responses to improve immunotherapy. On the other hand, ICI fails to work in not hot tumors due to the lack or even absence of pre-existing immunity [8]. Separating not hot tumors from hot tumors would benefit patient prognosis and may be helpful for clarifying immunotherapy-resistance mechanisms. Considering gene expression as a good indicator in immunotherapy prediction, we used UC gene expression to divide patients into two groups by the immune-based notion of hot tumors for immunotherapy [6, 8]. Subsequently, patients were subdivided according to the risk scores for exploration of combination immunotherapy.

In our study, we utilized UC metabolism characterization to perform a consensus clustering analysis and build a risk model. Through subdivision, each patient was categorized into two tags, clusters, and risk groups for individualized therapy and precision medicine. This study may not only spark new ideas in immunotherapy but also bridge the gap between bioinformatics analyses and clinical practice.

## Materials and Methods

**Fig. 1:**
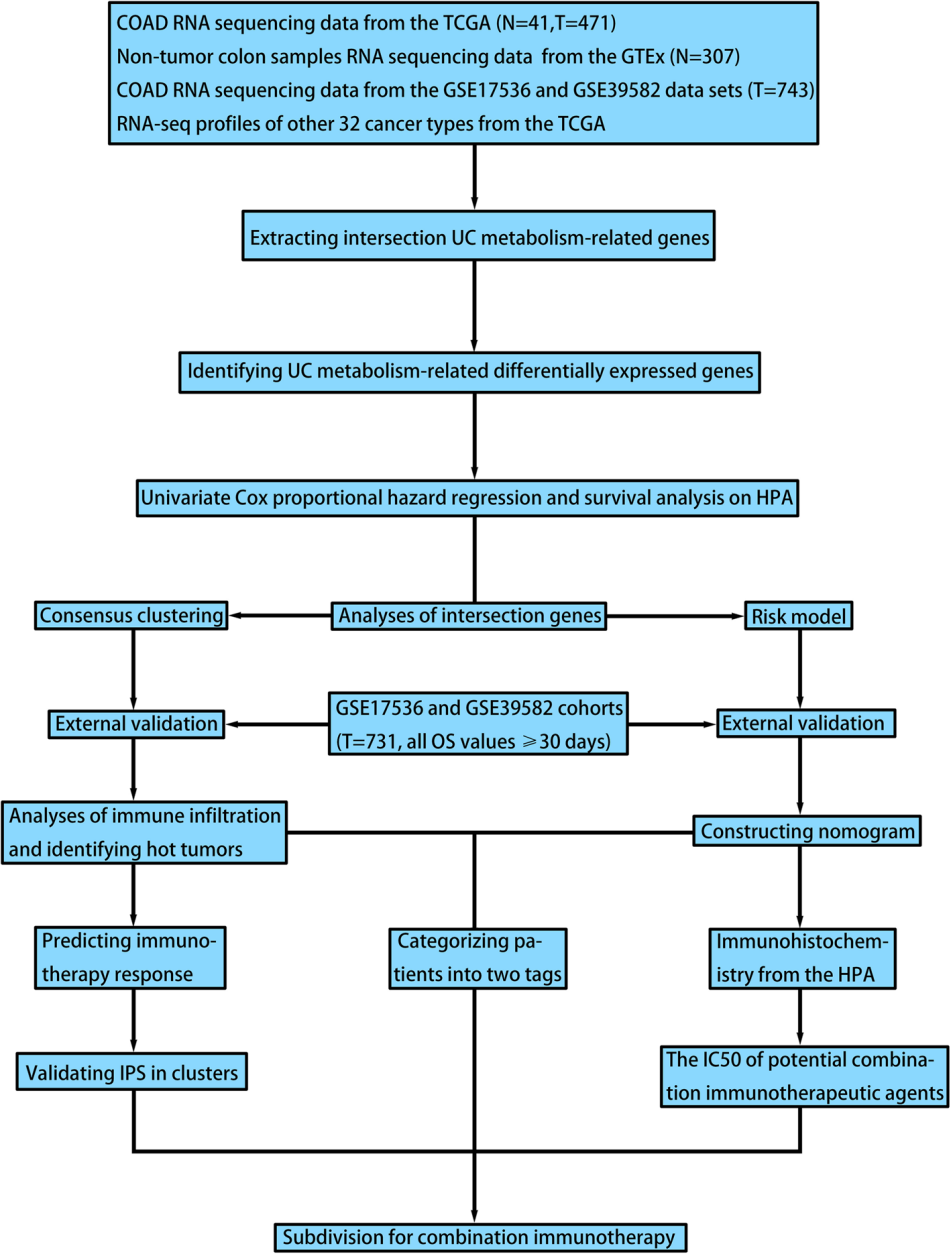
Flowchart of the study

The study design and procedure were visually represented as a flowchart (Fig. 1).

### Data preparation

The RNA-seq profiles of 471 colon cancer (COAD) and 41 non-tumor samples from The Cancer Genome Atlas (TCGA) as well as 307 non-tumor colon samples from the Genotype-Tissue Expression Project (GTEx) were downloaded from the University of California Santa Cruz (UCSC) webpage and the GTEx, respectively [14]. Their counts format profiles were used to identify differentially expressed genes (DEGs) [15]. Moreover, RNA-seq profiles of another 32 cancer types were obtained from the UCSC. Subsequently, we used public R packages to perform bioinformatic analyses. All public R packages were downloaded from the comprehensive R archive Network (CRAN) (https://cran.r-project.org/) and Bioconductor (http://www.bioconductor.org/). Fragments per kilobase million (FPKM) format RNA-seq profiles of 33 cancer types were converted into TPM (transcripts per million) for further analyses using the limma R package. We also obtained their clinical data on overall survival (OS), copy number segments and TMB from the UCSC and TCGA (v30.0 September 23, 2021). Similar information was downloaded from the GSE17536 and GSE39582 datasets for external validation from Gene Expression Omnibus (GEO) [16, 17]. To reduce statistical bias, samples with missing OS or short OS values (<30days) were excluded from all cohorts. Moreover, samples which had received immunotherapy should be excluded. According to clinical information, no patients had received immunotherapy. As a result, we obtained 424 colon cancer patients from the TCGA cohort and 731 patients from the external validation cohort.

### Identification and analyses of UC metabolism-related DEGs

UC metabolism-related genes with a relevance score of >9 were extracted from the GeneCards database [18–20]. We identified their intersection genes in the TCGA, GTEx, GSE17536, and GSE39582 cohorts using the VennDiagram R package. We used the counts format RNA-seq profiles to screen DEGs using the limma R package [15]. Intersection genes with an absolute value of log_2_ fold change >0.5 and a false discovery rate (FDR) adjusted *p*-value < 0.05, were identified as UC metabolism-related DEGs, followed by visualization of the volcano plot using the ggplot2 R package. A univariate Cox proportional hazard regression was performed to identify genes with a prognostic value (*p* < 0. 05) from the DEGs in the TCGA cohort [21]. A survival analysis of prognostic genes was performed for validation using the Human Protein Atlas (HPA). The HPA information was available from v103.38.proteinatlas.org and their URL was listed in the supplementary materials for intellectual property rights protection. Genes that had potent prognostic value not only in our study but also on HPA were called intersection prognostic genes. Genes that had potent prognostic value, not only in our study but also in the HPA, were called intersection prognostic genes. We analyzed the correlation of the expression of the genes with immune infiltration level using the Tumor IMmune Estimation Resource (TIMER) database [22]. The protein-protein interactions (PPI) and copy number variation (CNV) frequencies were also displayed using the STRING and TIMER databases. All data and download files in STRING are freely available under a 'Creative Commons BY 4.0' license. We located them in human chromosomes using the RCircos R package and compared infiltration levels among tumors with different somatic copy number for these intersecting prognostic genes [23].

### Consensus clustering analysis

To identify intrinsic groups sharing biological characteristics, intersecting prognostic genes were used to find the ‘cleanest’ cluster partition, where items nearly always cluster together, indicating a high consensus in colon cancer using the ConsensusClusterPlus R package [24]. Using the intersection prognostic genes, we performed a survival analysis, T-distributed stochastic neighbor embedding (t-SNE), and principal component analysis (PCA) of the clusters using the survminer, Rtsne and scatterplot3d R packages. Similar analyses were performed for the intersection prognostic genes in the external validation cohort.

### Immune characteristics and ICI

To explore the potential functions and pathways of the specific cluster, we used gene set enrichment analyses (GSEA) software and curated gene sets (kegg. v7. 4. symbols. gmt). The results are displayed in multi-GSEA diagrams using the ggplot2 and gridExtra R packages. We then calculated the immune infiltration of each colon cancer sample using CIBERSORT (R scrip v 1.03), estimated immune-based scores, GSVA and GSEABase R packages, and checkpoint expression using reshape2 and ggpubr R packages [25]. Subsequently, they were illustrated in a heatmap, violin plots, bubble chart and boxplots. To predict patient response to immunotherapy, we decided to use an IPS as the predictor. The IPS is an aggregated score based on the expression of representative genes or gene sets comprising four categories: MHC molecules, immunomodulators, effector cells (such as CD8^+^ T cells), and suppressor cells (Tregs and MDSCs). From The Cancer Immunome Atlas (TCIA), We obtained the aggregated score in the TCGA cohort from TCIA, and then visualized them using violin plots [26]. The data are published under a Creative Commons BY 4.0' license, attributed to the TCIA.

### TMB and microsatellite instability (MSI)

TMB data of 33 cancer types in the “Masked Somatic Mutation” type processed by VarScan2, were extracted from the TCGA [27]. We analyzed the correlation of intersection prognostic genes expression with TMB levels in multiple cancers and visualized them as radar charts using the fmsb R package. Waterfall plots and boxplots of TMB in clusters were also illustrated using maftools and ggplot2 R packages. In addition, in our study we visualized the MSI status of clusters in the TCGA cohort for further validation [28].

### Risk model and nomogram

We randomly divided half of the colon cancer cases in the TCGA cohort into training and testing sets. Next, we performed a LASSO regression analysis and generated a risk model by controlling the first-rank value of Log(λ) at the minimum likelihood of deviance using caret and glmnet R packages [21, 25]. The risk score for each colon cancer patient was calculated using the following formula:

$$\mathrm{Risk \; score}=\sum_{\mathrm{k}=1}^{\mathrm{n}}{\mathrm{coefficient } \; (\mathrm{gene }}^{\mathrm{k}}) *\mathrm{ expression } \; {(\mathrm{gene }}^{\mathrm{k}})$$

In the training, testing, and complete sets, we visualized risk scores, status, survival analyses, and receiver operating characteristic (ROC) curves of colon cancer patients using the survival, survminer, and timeROC R packages. Risk scores for the samples in the external validation cohort were also calculated using the same formula and survival analysis [21].

Based on the clinical data of colon cancer patients, we performed univariate Cox and multivariate Cox regression analyses to identify independent prognostic factors and established a nomogram using the survival, rms, and regplot R packages. The ROC curves and calibration plots of the nomogram were displayed for evaluation using the timeROC and nomogramEx R packages.

### Exploration in subdividing

We analyzed the correlation of clusters and MSI status with risk scores and visualized them as bar plots and box plots using plyr and ggplot2 R packages. A survival analysis was performed after subdividing colon cancer patients into the TCGA cohort. Subsequently, we illustrated a Sankey diagram of clusters, risk groups, and MSI status of patients with colon cancer using the ggalluvial R package. The half-maximal inhibitory concentration (IC50) of potential combination immunotherapeutic agents in colon cancer samples in the TCGA cohort was predicted using the pRRophetic R package [25].

### Intersection prognostic genes on HPA

For further validation, several intersection prognostic genes immunohistochemistry images were displayed from the HPA. These HPA images were available at v103.38.proteinatlas.org and their URL had been mentioned in the figure legend to protect the developers' intellectual property rights.

## Results

### Identification and analyses of UC metabolism-related genes for clusters

**Fig. 2:**
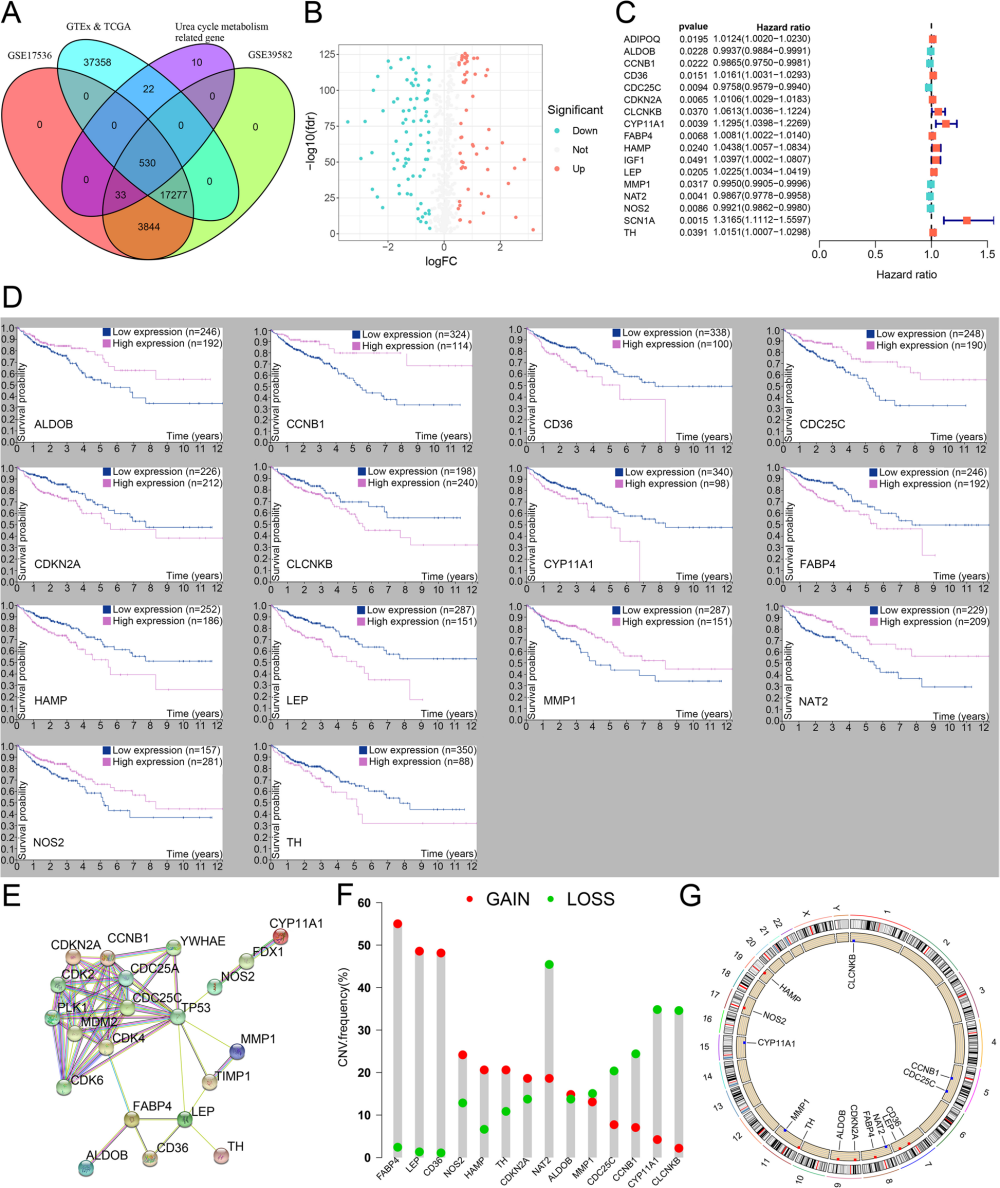
Identification and analyses of urea cycle (UC) metabolism-related genes. **A** The Venn diagram of UC metabolism-related genes, TCGA, GTEx, GSE17536, and GSE39582 data sets. **B** The volcano plot of 132 differentially expressed UC metabolism-related genes. **C** The forest plot of prognostic UC metabolism-related genes in our univariate Cox proportional hazard regression. **D** 14 UC metabolism-related genes Kaplan–Meier survival curves of OS from HPA (All *p*-value < 0.05 in Kaplan–Meier survival curves). **E** The PPI of 14 UC metabolism-related genes. **F** The CNV frequency of prognostic 14 UC metabolism-related genes. **G** The CNV of 14 UC metabolism-related genes on RCircos 2D track plot with human genome

We extracted 565 UC-related genes in colon cancer from GeneCards (Additional file 5: Appendix T1). Of these, 530 were considered to be intersecting genes in colon cancer (Fig. 2A). There were 77 downregulated DEGs and 55 upregulated DEGs (Fig. 2B). Of these, 17 DEGs may have prognostic values (Fig. 2C). For validation, we listed the results of their survival analyses (Additional file 6: Appendix T2). We identified 14 DEGs with potent prognostic values and displayed Kaplan-Meier survival curves of OS (Fig. 2D). The results found that *CD36*, *CDKN2A*, *CLCNKB*, *CYP11A1*, *FABP4*, *HAMP*, *LEP* and *TH* were upregulated in colon cancer while the others were downregulated. Almost all 14 intersection prognostic genes were associated with immune infiltration levels in colon cancer, including *CCNB1* and *CD36* (*p* < 0. 05) (Additional file 1: Appendix D1, Additional file 4: Figure S1A). In addition, we found that some of genes (such as *CCNB1*, *NOS2* and *LEP*) might be associated with *TP53* (Fig. 2E). Of these, seven intersection prognostic gene frequencies of gain CNV were higher than that of the loss and was marked in red on the 2D track plot, while the others of gain CNV were lower than the loss and was marked in blue (Fig. 2F-G). Furthermore, we found that almost all intersection prognostic genes’ alterations in the copy number were correlated with the infiltration level of CD8^+^ T cells (*p*<0.05, Additional file 4: Figure S1B).

### Clusters and external validation in colon cancer

**Fig. 3:**
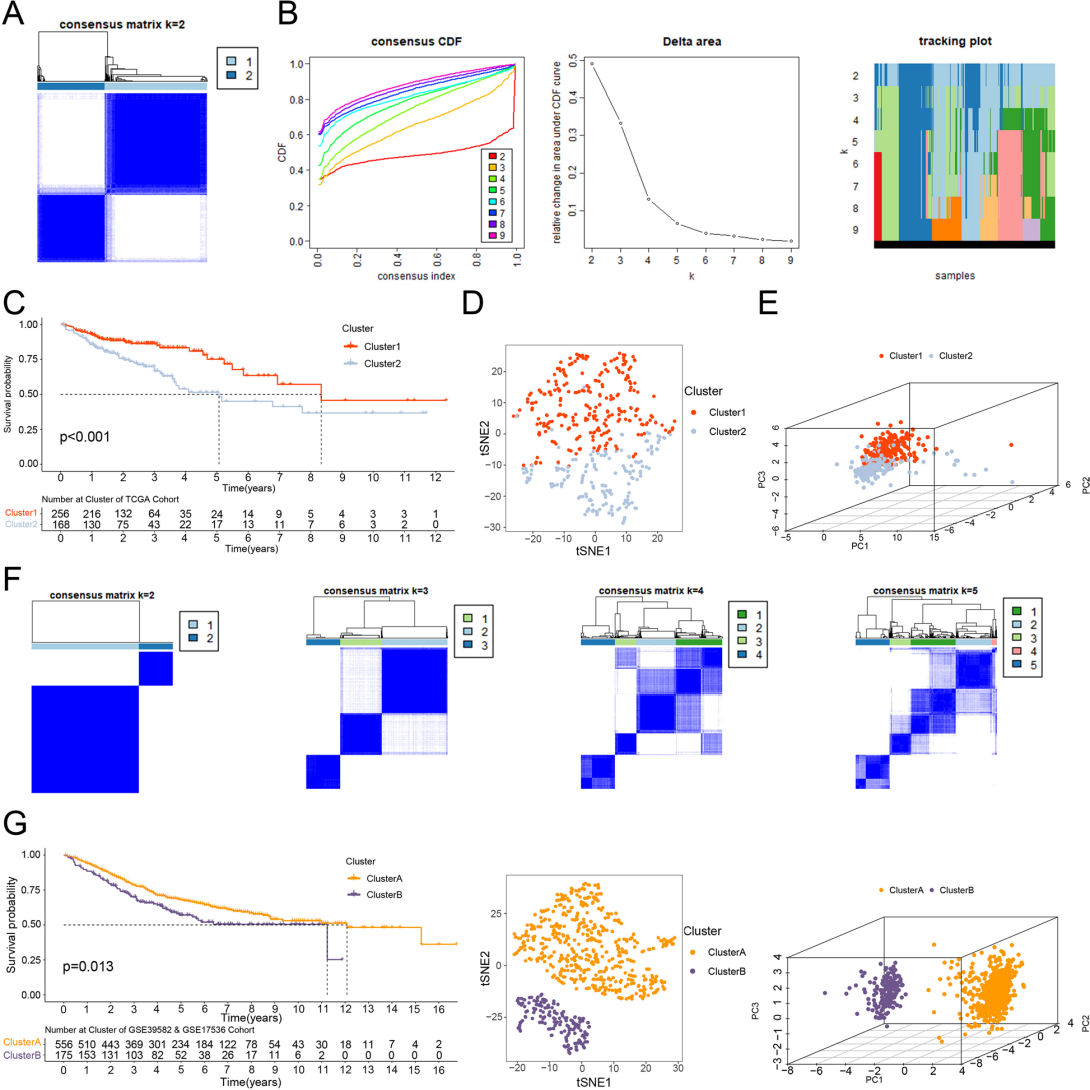
Consensus clustering analysis. **A** The consensus matrix k = 2 in the TCGA cohort. **B** The CDF plots and the item tracking plot of consensus clustering matrix in the TCGA cohort. **C** Kaplan–Meier survival curves of OS in clusters. **D**, **E** The t-SNE and 3D PCA separated two clusters of colon cancer patients in the TCGA cohort. **F** The consensus matrix k = 2, k = 3, k = 4, and k = 5 in the external validation cohort. **G** Kaplan–Meier survival curves of OS in clusters, the t-SNE and 3D PCA separated two clusters of colon cancer patients in the external validation cohort

According to the consensus clustering analysis with 14 intersection prognostic genes, the ‘cleanest’ cluster partition was k=2 and is based on the cumulative distribution function (CDF) plots and the item tracking plot (Fig. 3A-B). The consensus matrix legend and other consensus matrices are also displayed (Additional file 4: Figure S2A). Samples in cluster 1 had a better OS than those in cluster 2 (Fig. 3C). The t-SNE and 3D PCA analyses clearly separated the cases in cluster 1 from the cluster, which further verified our cluster distribution (Fig. 3D-E). The ‘cleanest’ cluster partition was also k=2 in the external validation cohort based on consensus clustering analysis (Fig. 3F, Additional file 4: Figure S2B). Similarly, colon cancer patients in cluster A had a better OS than those in cluster B and were clearly separated from cluster B in the t-SNE and 3D PCA analyses (Fig. 3G).

### Immune landscapes and ICI of clusters

**Fig. 4:**
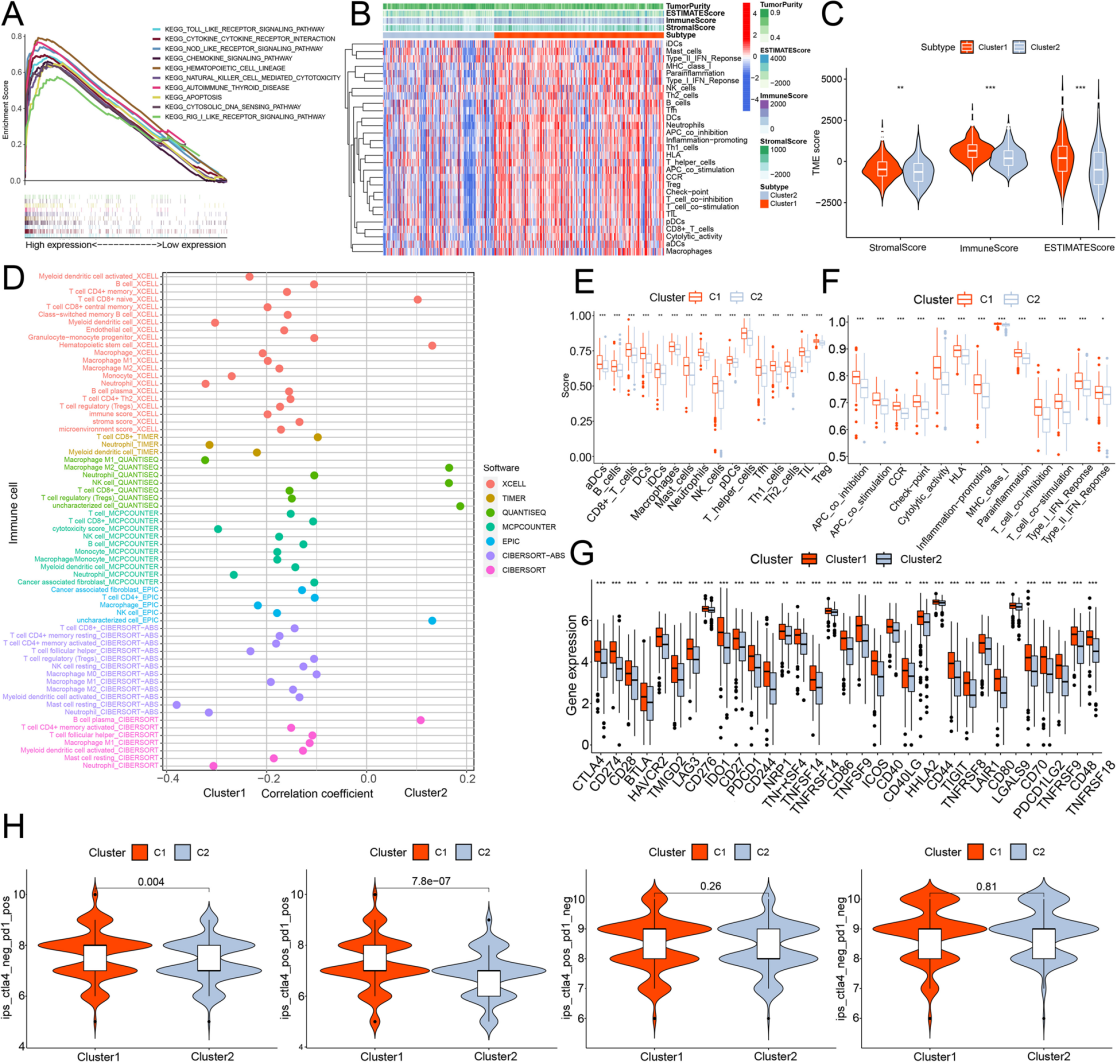
Tumor immune characteristics and ICI in clusters of the TCGA cohort. **A** The multi-GSEA of colon cancer in the cluster 1. **B** The heatmap of immune infiltration between two clusters in colon cancer. **C** The violin plots of immune-based scores in clusters (* means *p* < 0.05, **p < 0.01 and ***p < 0.001). **D** The correlation coefficient of immune infiltration between two clusters of colon cancer cases. **E**, **F** The single sample GSEA immune infiltration and immune related functions between two clusters (* means *p* < 0.05, **p < 0.01 and ***p < 0.001). **G** 33 checkpoints’ expression level between two clusters (* means *p* < 0.05, **p < 0.01 and ***p < 0.001). (H) The IPS of *PD-1* and/or *CTLA4* between two clusters of colon cancer cases

The GSEA indicated that the biological functions of cluster 1 were associated with immunity, as most of the top 10 enriched pathways were correlated with immune functions, such as chemokine signaling (all *p* < 0. 01, FDR <0. 01, the absolute value normalized enrichment score >2.1; Fig. 4A, Additional file 4: Figure S2C) [29]. Colon cancer patients in the cluster 1 had immune cell infiltrates, lower tumor purity, and higher immune-related scores (Fig. 4B-F, Additional file 2: Appendix D2). In addition, cases in the cluster 1 had higher 33 checkpoints’ expression than the cluster 2 in the TCGA cohort such as *CTLA4*, *HAVCR2*, *LAG3* and *PDCD1*(all *p*<0.05, Fig. 4G). We found that patients in cluster 1 showed significantly higher IPS for *PD-1* ICI as well as *PD-1 and CTLA4* ICI (all *p* < 0.05). However, there were no significant differences in other ICI between clusters 1 and 2 (Fig. 4H).

### Analyses of TMB and MSI of clusters

**Fig. 5:**
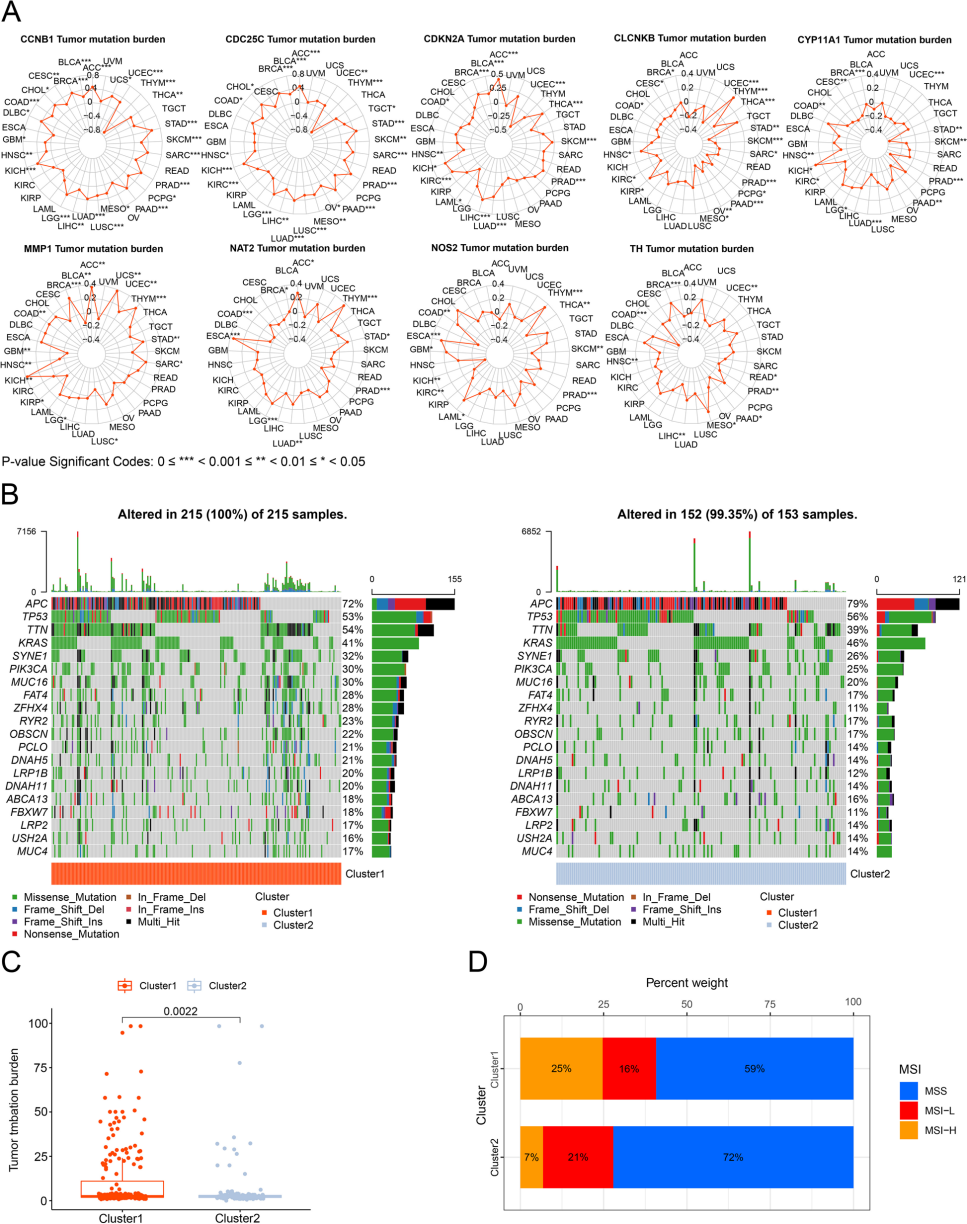
TMB and MSI of clusters for validation. **A** Radar chart of nine genes on TMB (* means *p* < 0.05, **p < 0.01 and ***p < 0.001, ACC: adrenocortical cancer, BLCA: bladder cancer, BRCA: breast cancer, CESC: cervical cancer, COAD: colon cancer, CHOL: bile duct cancer, DLBC: large B-cell lymphoma, ESCA: esophageal cancer, GBM: glioblastoma, HNSC: head and neck cancer, KICH: kidney chromophobe, KIRC: kidney clear cell carcinoma, KIRP: kidney papillary cell carcinoma, LAML: acute myeloid leukemia, LGG: lower grade glioma, LIHC: liver cancer, LUAD: lung adenocarcinoma, LUSC: lung squamous cell carcinoma, MESO: mesothelioma, OV: ovarian cancer, PAAD: pancreatic cancer, PCPG: pheochromocytoma & paraganglioma, PRAD: prostate cancer, READ: rectal cancer, SARC: sarcoma, SKCM: melanoma, STAD: stomach cancer, TGCT: testicular cancer, THCA: thyroid cancer, THYM: thymoma, UCEC: endometrioid cancer, UCS: uterine carcinosarcoma, UVM: andocular melanomas). **B** Waterfall plot of TMB in clusters. **C** The TMB level between two clusters of colon cancer cases. **D** The percentage weight of MSI status between two clusters in the TCGA cohort

Nine intersection prognostic genes were related to TMB in colon cancer and were visualized as radar charts. The number represents the correlation coefficient of TMB in diverse cancer types (Fig. 5A, Additional file 3: Appendix D3). The waterfall plots showed the TMB of individual patients in clusters 1 and 2 (Fig. 5B). The TMB in cluster 1 was higher than that in cluster 2, indicating better immunotherapy (*p*<0.05, Fig. 5C). Patients in cluster 1 had a higher percentage weight of MSI-H, whereas cluster 2 had a higher ratio of MSI-L and microsatellite-stable (MSS) (Fig. 5D).

### Risk model and external verification

**Fig. 6:**
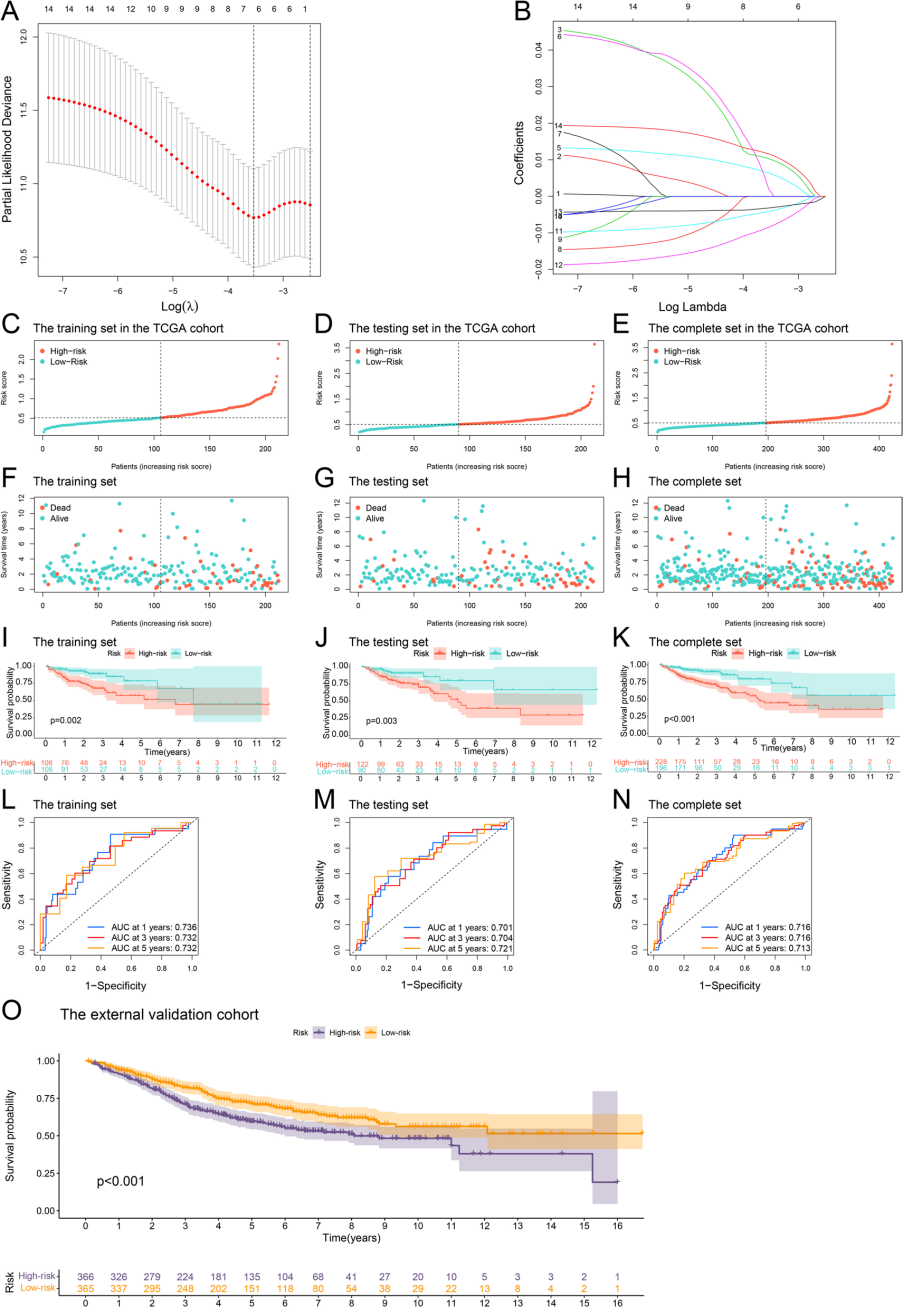
Risk model and external validation. **A**, **B** Constructing a risk model of seven genes model by LASSO regression. **C**, **D**, **E** Risk scores of colon cancer patients in the training, testing, and complete sets, respectively. **F**, **G**, **H** Survival time and survival status of colon cancer cases between low- and high-risk groups in the train, testing, and complete sets in the TCGA cohort. **I**, **J**, **K** Kaplan–Meier analysis of OS between low- and high-risk groups in the training, testing and complete sets in the TCGA cohort. **L**, **M**, **N** The ROC curves for 1-, 3- and 5-year OS of colon cancer samples in the training, testing and complete sets in the TCGA cohort. **O** Kaplan–Meier analysis of OS in the external validation cohort

We built a risk model of seven genes when the first-rank value of Log(λ) was at the minimum likelihood (Fig. 6A-B). The risk score formula was *CD36* × 0.0103 + *CDKN2A* × 0.0064 + *CLCNKB* × 0.0019 + *MMP1* × (-0.0041) + *NAT2* × (-0.0084) + *NOS2* × (-0.0032) + *TH* × 0.0116. The cut-off risk score was 0.5, and patients were subdivided into low- and high-risk groups in the training, testing, and complete sets of cases (Fig. 6C-E). Their survival status and survival time were showed (Fig. 6F-H). According to the Kaplan-Meier survival curves of OS, we proved that patients in the high-risk group had a worse prognosis (all *p*<0.01, Fig. 6I-K). The area under the curve (AUC) for 1-, 3-, and 5-year OS was all > 0.700 in the training, testing, and complete sets of cases (Fig. 6L-N). Colon cancer patients in the low-risk group were clearly separated from those in the high-risk group in the t-SNE and 3D PCA analyses (Additional file 4: Figure S2D-E). Meanwhile, patients in the high-risk group exhibited a worse OS in the external validation cohort, indicating successful external verification (*p*<0.001, Fig. 6O).

### Generation and assessment of Nomogram

**Fig. 7:**
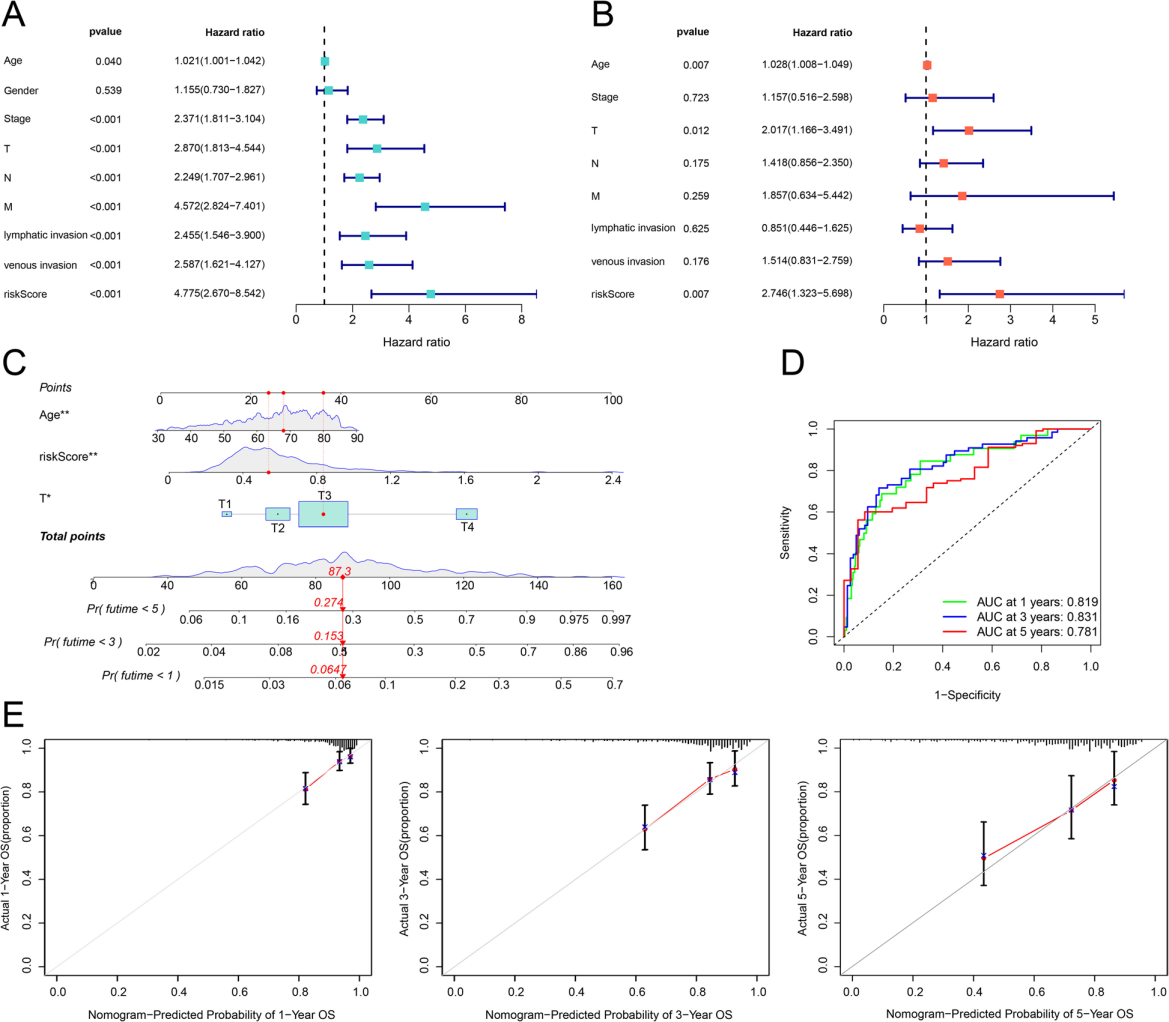
Generation and assessment of Nomogram. **A**, **B** Identifying independent risk factors of clinical characteristics for worse OS by univariate and multivariate Cox regression. **C** Nomogram based on age, tumor T stage, and risk scores. **D** The ROC curves for 1-, 3- and 5-year OS of colon cancer patients in the nomogram. **E** The calibration curves for 1-, 3- and 5-year OS

According to results of the univariate Cox (uni-Cox) regression and the multivariate Cox (multi-Cox), we considered patients’ age, tumor T stage, and risk score as independent risk factors with a prognostic value and generated a nomogram with these independent risk factors in colon cancer (Fig. 7A-C). The AUC of the nomogram for 1-, 3-, and 5-year OS was all > 0.780 in the TCGA cohort (Fig. 7D). Similarly, the calibration plots of the nomogram for 1-, 3-, and 5-year OS were consistent with the predictions (Fig. 7E).

### Subdividing for combination immunotherapy

**Fig. 8:**
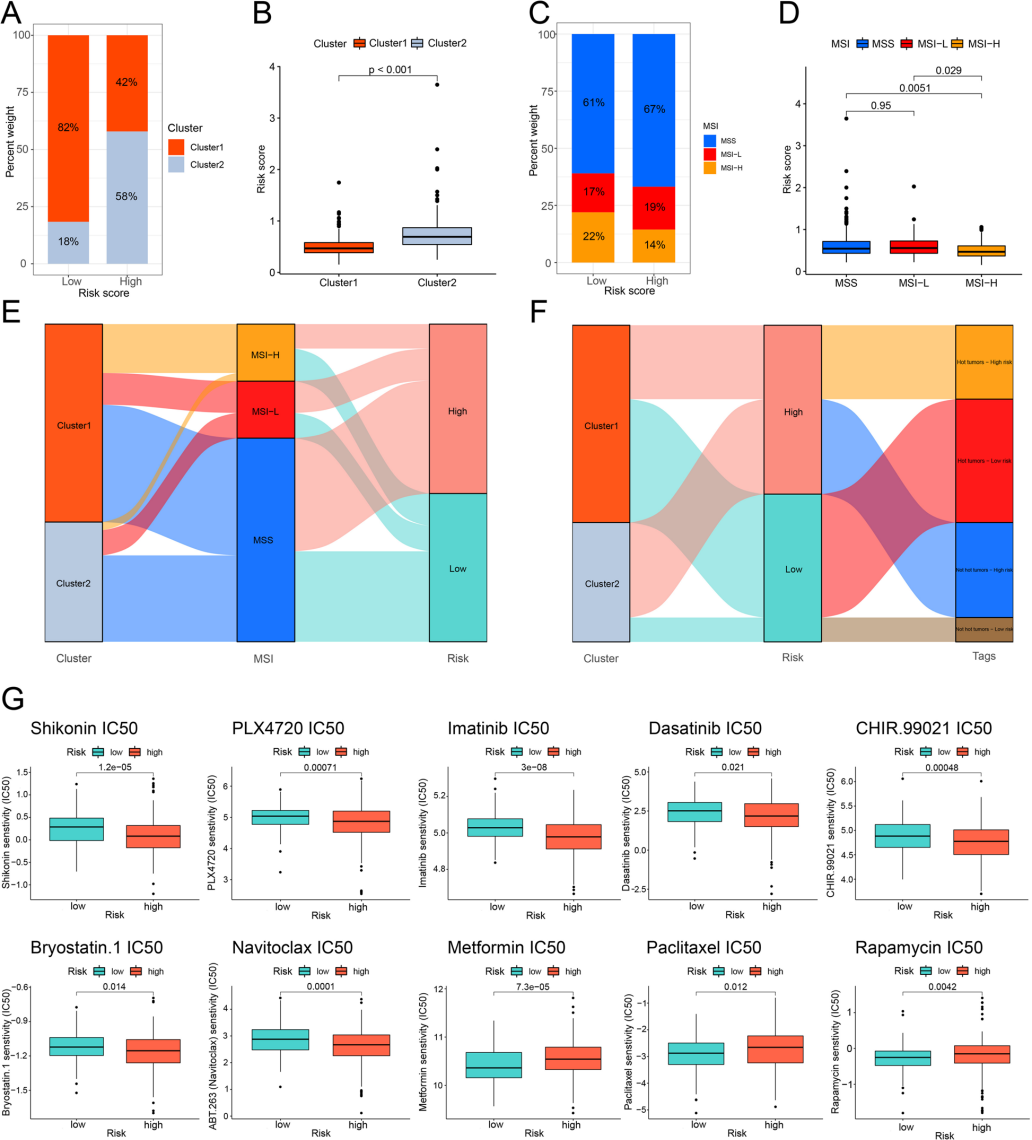
Subdividing colon cancer cases for combination immunotherapy. **A** The percentage weight of clusters between low- and high-risk groups in the TCGA cohort. **B** The levels of risk score in two clusters of colon cancer. **C** The percentage weight of MSI status between low- and high-risk groups in the TCGA cohort. **D** The levels of risk score in different MSI status. **E** The Sankey diagram of clusters, risk groups and MSI status of colon cancer cases. **F** The Sankey diagram of clusters, risk groups and tags of colon cancer cases. **G** The prediction of potential combination immunotherapeutic agents

The percentage weight of cluster 1 in the low-risk group was higher than that in the high-risk group, and patients in cluster 1 had a lower risk score than those in cluster 2, which was consistent with our survival analyses (*p*<0.001, Fig. 8A-B). The percentage weight of MSI-H was 22% in the low-risk group and 14% in the high-risk group (Fig. 8C). The risk score of patients with MSI-H status was lower than that of patients with MSI-L status (*p*=0.029) and MSS status (*p*=0.0051, Fig. 8D). The Sankey diagram showed the connection among patients in different clusters, risk groups, and MSI status (Fig. 8E). The Sankey diagram also showed the connection among patients in different clusters, risk groups and tags (Fig. 8F). Furthermore, we calculated the IC50 in ten potential combination immunotherapeutic agents for individual patients in the TCGA cohort. The IC50 of seven agents for patients in the high-risk group were lower than that in the low-risk group, such as shikonin. Meanwhile, patients in the low-risk group might be more sensitive to other drugs, such as metformin (Fig. 8G).

### Immunohistochemistry

We examined the histological expressions of four intersection prognostic genes with significant differences in the protein levels between the normal colon and cancerous colon tissues. The protein expression of *CDKN2A* and *CLCNKB* were upregulated in the cancerous colon tissues while that of others were downregulated or even undetectable in cancerous colon tissues (Additional file 4: Figure S3A-B).

## Discussion

As the most rapidly growing drug class, immunotherapies may be considered promising anticancer therapeutic interventions [8]. However, it is essential to stratify patients because of the limited population benefits of immunotherapy [6]. In our study, colon cancer patients were effectively and reliably distinguished into clusters 1 and 2. Next, the patients were subdivided into low- and high-risk groups. Each case was categorized into two tags, clusters, and risk groups for individual combination immunotherapy.

Reprogramming of cellular metabolism is a hallmark of cancer cells. Metabolic alterations increase the demand for nitrogen and regulated UC metabolism, which had an influence on gene expression and immune infiltration [9, 10]. Tumor immune infiltration is associated with anticancer therapeutic interventions [8]. To guide decisions on therapy, we established the notion of hot tumors, which refer to T cell-infiltrated and inflamed tumors. Owing to the pre-existing immunity in hot tumors, ICI can disrupt tolerance to help CD8^+^ T cells target cancer cells by releasing perforin, granulysin, and granzymes [8]. Their characteristics include high IPS and active checkpoint expression, such as *PD-1*, *CTLA4*, *TIME3* and *LAG3*. Referring to the association of UC metabolism with immune infiltration and immunotherapy, we attempted to use 14 UC metabolism-related genes to stratify colon cancer patients [6, 10, 11]. All of them had a potent prognostic value and their expression was considerably related to CD8^+^ T cell infiltration. Based on these unique genes, we stratified the patients into two clusters to identify hot tumors. Immune infiltration analyses revealed that colon cancer in cluster 1 was characterized by higher immune scores, higher immune infiltration levels (e.g., CD8^+^ T cells), increased active immune function (e.g., inflammatory promotion and cytolytic activity), and overexpression of immune checkpoints such as *CTLA4*, *TIME3* (*HAVCR2*), *LAG3,* and *PD-1* (*PDCD1*) than in cluster 2 [8]. The IPS is a scoring scheme for the quantification and is super as a predictor of ICI. Its AUC is higher than 0.99 [26]. The TMB and MSI status of the two clusters were also displayed for validation. Colon cancer in cluster 1 displayed significantly higher *PD-1* and *CTLA4* IPS, and showed significantly higher levels of TMB and ratio of MSI-H [6, 30]. Above all, we can effectively identify hot tumors for colon cancer immunotherapy.

In addition, changing not hot tumors into hot tumors before immunotherapy would significantly help immunotherapy. Some agents can act as adjuvants or even induce tumor infiltration by immune cells to improve immunotherapy, such as metformin [8, 31]. According to the results of the KEYNOTE-189 clinical trial, patients derive benefits after the addition of ICI to the standard chemotherapy [7]. Consequently, we built a risk model to calculate the risk score of each sample and subdivided it to explore combination immunotherapy. Colon cancer patients in the high-risk group were more sensitive to seven potential combination immunotherapeutic agents such as shikonin and dasatinib [32, 33]. Furthermore, the low-risk group displayed lower IC50 of metformin, paclitaxel and rapamycin [31, 34, 35]. Eventually, we were able to subdivide them into four groups with two tags, the cluster 1 + low-risk group, the cluster 1 + high-risk group, the cluster 2 + low-risk group and the cluster 2 + high-risk group, and assist in the rational combination immunotherapy. Combination immunotherapy may improve clinical treatment and reduce adverse effects in hot tumors. For not hot tumors, we could also improve immunotherapeutic efficacy or even develop new therapeutic strategies to change them into hot tumors, changing the dilemma of immunotherapy because it is feasible that chemical agents could promote immune cell infiltration into the tumor environment [31–36].

In our study, improved stratification was achieved by using 14 UC metabolism-related genes. This will contribute to elucidating immunotherapy resistance mechanisms and improving precision medicine. To further validate our stratification, external validation was performed on the consensus clustering analysis and risk model of the GSE39582 and GSE17536 cohorts. Patients with colon cancer may benefit from our stratification. An immunohistochemistry analysis of four genes was displayed. Of these, the frequency of *CDKN2A* methylation in MSI-L colon tumors is less than that in MSI-H or MSS tumors and might be associated with poor prognoses [37]. The infiltration of *NOS2*-positive macrophages cells correlates to a comparable beneficial prognostic effect in stage I-II colon cancers [38]. However, the functions of other genes require exploration.

There were several limitations in our study. There was a lack of in vivo or in vitro experiments to explore the molecular function of urea cycle metabolism-related genes. A rigorous experimental design and extensive experiments would be helpful in clarifying the mechanisms of these genes and their association with immunotherapy. As this was a retrospective study, potential bias could not be ruled out. In addition, although several independent datasets were used for external validation, we did not validate our results in clinical practice. Therefore, prospective and large-scale studies should be designed to validate our study further. Our study will be helpful in bridging the gap between bioinformatics and clinical research. In the future, it may be likely that specific bioinformation of patients may be analyzed using different methods; further, patients may be categorized into more distinct rational clinical tags (e.g., clusters and risk groups) for personalized cancer treatment. This study is worth exploring as a starting point of this promising field.

## Conclusions

Our study showed that UC metabolism characterization could contribute to subdividing patients with colon cancer for combination immunotherapy. In addition, it demonstrated that bioinformation can be used to provide additional clinical information of patients through improved stratification. By subdividing patients, more rational information on clinical clusters and risk groups can be established to improve the precision of medication. However, there is still a long way to go to improve combination immunotherapy.

## Supplementary Information

**Additional file 1: Appendix D1. **The correlation of 14 UC metabolism-related geneexpression with immune infiltration level in colon cancer.

**Additional file 2: Appendix D2. **The immune infiltrates inclusters in the TCGA cohort.

**Additional file 3: Appendix D3.** The correlation of nine UCmetabolism-related gene expression with TMB in diverse cancer types.

**Additional file 4: Appendix S1-S3. Figure S1.** The association of intersection prognostic genes with immunity. (A) The correlation of 14 UC metabolism-related genes expression level with immune infiltration level in colon cancer. (B) Comparisons of immune infiltration level among samples with copy number alteration and the normal of 14 UC metabolism-related genes. **Figure S2.** The consensus clustering analysis of clusters, GSEA of the Cluster 1, and the t-SNE and 3D PCA of risk groups in the TCGA cohort. (A) The consensus matrix legend and the other consensus matrixes of clusters in the TCGA cohort. (B) The CDF plots and the item tracking plot of consensus clustering matrix in the external validation cohort. (C) The GSEA of cluster 1 colon cancer in the TCGA cohort. (D) (E) The t-SNE and 3D PCA of risk groups in the TCGA cohort. **Figure S3.** Immunohistochemistry of four genes in normal colon tissue and cancerous colon tissue, respectively. (A) The protein expression of upregulated genes (*CDKN2A* and *CLCNKB*) in normal colon tissue and cancerous colon tissue respectively. The URL of *CDKN2A* was https://www.proteinatlas.org/ENSG00000147889-CDKN2A/tissue/colon#img,https://www.proteinatlas.org/ENSG00000147889-CDKN2A/pathology/colorectal+cancer#img. The URL of *CLCNKB* was https://www.proteinatlas.org/ENSG00000184908-CLCNKB/tissue/colon#img and https://www.proteinatlas.org/ENSG00000184908-CLCNKB/pathology/colorectal+cancer#img. (B) The protein expression of downregulated genes (*NAT2* and *NOS2*) in normal colon tissue and cancerous colon tissue respectively. The URL of *NAT2 *was https://www.proteinatlas.org/ENSG00000156006-NAT2/tissue/colon#img and https://www.proteinatlas.org/ENSG00000156006-NAT2/pathology/colorectal+cancer#img. The URL of *NOS2 *was https://www.proteinatlas.org/ENSG00000007171-NOS2/tissue/colon#img and https://www.proteinatlas.org/ENSG00000007171-NOS2/pathology/colorectal+cancer#img.

**Additional file 5: Appendix T1. **The urea cyclemetabolism-related genes.

**Additional file 6: Appendix T2. **The survival analysis ofprognostic genes on HPA for validation.

## Data Availability

The data used to support the results are available from the UCSC (http://xena.ucsc.edu/), TCGA(https://portal.gdc.cancer.gov/), GEO (https://www.ncbi.nlm.nih.gov/geo/), CRAN (https://cran.r-project.org/) and Bioconductor (http://www.bioconductor.org/). HPA (https://www.proteinatlas.org/), the Tumor IMmune Estimation Resource (TIMER) database (https://cistrome.shinyapps.io/timer/), STRING (https://cn.string-db.org/cgi/input.pl), GSEA (http://www.gsea-msigdb.org/gsea/index.jsp), and TCIA (https://tcia.at/home).
