# Supplementary material for: Patient stratification based on urea cycle metabolism for exploration of combination immunotherapy in colon cancer
Source: BMC Cancer. 2022 Aug 13;22:883. doi: 10.1186/s12885-022-09958-7 (PMC9375340; doi:10.1186/s12885-022-09958-7)
Supplement: Supplementary file 1 — Additional file 1: Appendix D1. The correlation of 14 UC metabolism-related geneexpression with immune infiltration level in colon cancer. [file 12885_2022_9958_MOESM1_ESM.docx]

## Supplementary Materials

**Additional file 1: Appendix D1**

Appendix D1: The correlation of 14 UC metabolism-related gene expression with immune infiltration level in colon cancer.

ID cancer variable partial.cor p

ALDOB COAD Purity -0.126600889 0.010572628

ALDOB COAD B Cell 0.114405993 0.021450296

ALDOB COAD CD8+ T Cell 0.034874259 0.483463113

ALDOB COAD CD4+ T Cell -0.015893256 0.750722691

ALDOB COAD Macrophage -0.029631975 0.552592164

ALDOB COAD Neutrophil 0.031759712 0.525974853

ALDOB COAD Dendritic Cell 0.036385565 0.466921216

CCNB1 COAD Purity 0.085847283 0.083671081

CCNB1 COAD B Cell 0.131439476 0.008164259

CCNB1 COAD CD8+ T Cell 0.177848952 0.00031668

CCNB1 COAD CD4+ T Cell -0.115879692 0.020126573

CCNB1 COAD Macrophage -0.041345775 0.407206665

CCNB1 COAD Neutrophil 0.19353885 9.60E-05

CCNB1 COAD Dendritic Cell 0.103813717 0.037470371

CD36 COAD Purity -0.325743166 1.63E-11

CD36 COAD B Cell 0.259151289 1.27E-07

CD36 COAD CD8+ T Cell 0.305118772 3.40E-10

CD36 COAD CD4+ T Cell 0.391297491 3.69E-16

CD36 COAD Macrophage 0.658055914 1.76E-51

CD36 COAD Neutrophil 0.500046129 9.30E-27

CD36 COAD Dendritic Cell 0.522260031 1.64E-29

CDC25C COAD Purity 0.186337607 0.000156311

CDC25C COAD B Cell 0.122092978 0.014063864

CDC25C COAD CD8+ T Cell 0.118505717 0.016899572

CDC25C COAD CD4+ T Cell -0.082172593 0.099926853

CDC25C COAD Macrophage -0.067691587 0.174487078

CDC25C COAD Neutrophil 0.099765377 0.045874403

CDC25C COAD Dendritic Cell 0.019974447 0.689687507

CDKN2A COAD Purity -0.066576833 0.180084867

CDKN2A COAD B Cell -0.112068916 0.024279428

CDKN2A COAD CD8+ T Cell -0.063044536 0.204920493

CDKN2A COAD CD4+ T Cell 0.126954386 0.010839895

CDKN2A COAD Macrophage 0.1331829 0.007349026

CDKN2A COAD Neutrophil 0.110324864 0.027167367

CDKN2A COAD Dendritic Cell 0.07577552 0.129331107

CLCNKB COAD Purity -0.10658705 0.031568911

CLCNKB COAD B Cell -0.126428151 0.010974351

CLCNKB COAD CD8+ T Cell -0.155059058 0.001726206

CLCNKB COAD CD4+ T Cell 0.213914362 1.52E-05

CLCNKB COAD Macrophage 0.181456845 0.000245971

CLCNKB COAD Neutrophil 0.046548112 0.352516365

CLCNKB COAD Dendritic Cell 0.076690977 0.124753676

CYP11A1 COAD Purity -0.177502813 0.000319951

CYP11A1 COAD B Cell 0.085976601 0.084356245

CYP11A1 COAD CD8+ T Cell 0.075917192 0.126716925

CYP11A1 COAD CD4+ T Cell 0.326433807 1.96E-11

CYP11A1 COAD Macrophage 0.365619648 3.19E-14

CYP11A1 COAD Neutrophil 0.176666986 0.000378397

CYP11A1 COAD Dendritic Cell 0.25321443 2.67E-07

FABP4 COAD Purity -0.357841726 9.70E-14

FABP4 COAD B Cell 0.085202812 0.087199256

FABP4 COAD CD8+ T Cell 0.179414683 0.000279618

FABP4 COAD CD4+ T Cell 0.347854228 7.07E-13

FABP4 COAD Macrophage 0.495432498 2.07E-26

FABP4 COAD Neutrophil 0.304695146 4.63E-10

FABP4 COAD Dendritic Cell 0.380338191 2.77E-15

HAMP COAD Purity -0.288110267 3.21E-09

HAMP COAD B Cell 0.016493365 0.741016277

HAMP COAD CD8+ T Cell 0.181796411 0.000230927

HAMP COAD CD4+ T Cell 0.223373228 6.13E-06

HAMP COAD Macrophage 0.458812586 2.00E-22

HAMP COAD Neutrophil 0.380859553 2.73E-15

HAMP COAD Dendritic Cell 0.417087729 2.37E-18

LEP COAD Purity -0.165680234 0.000792471

LEP COAD B Cell 0.051970413 0.297383837

LEP COAD CD8+ T Cell 0.189310201 0.000124298

LEP COAD CD4+ T Cell 0.243617665 7.65E-07

LEP COAD Macrophage 0.433543596 6.07E-20

LEP COAD Neutrophil 0.332152574 8.77E-12

LEP COAD Dendritic Cell 0.360546976 8.73E-14

MMP1 COAD Purity -0.281641772 7.39E-09

MMP1 COAD B Cell -0.040081054 0.421717037

MMP1 COAD CD8+ T Cell 0.242318309 7.76E-07

MMP1 COAD CD4+ T Cell 0.012552877 0.801883256

MMP1 COAD Macrophage 0.156845066 0.001564889

MMP1 COAD Neutrophil 0.514594637 1.71E-28

MMP1 COAD Dendritic Cell 0.345230405 1.08E-12

NAT2 COAD Purity 0.075973412 0.125966196

NAT2 COAD B Cell 0.166470064 0.000781947

NAT2 COAD CD8+ T Cell 0.110015729 0.026646789

NAT2 COAD CD4+ T Cell 0.048753034 0.329544705

NAT2 COAD Macrophage -0.067928409 0.172980852

NAT2 COAD Neutrophil -0.117248351 0.018840434

NAT2 COAD Dendritic Cell -0.028876945 0.563737856

NOS2 COAD Purity -0.131400128 0.007948198

NOS2 COAD B Cell 0.120360572 0.015498021

NOS2 COAD CD8+ T Cell 0.05710499 0.250959049

NOS2 COAD CD4+ T Cell 0.023899596 0.632819957

NOS2 COAD Macrophage -0.208560432 2.38E-05

NOS2 COAD Neutrophil 0.219553318 9.12E-06

NOS2 COAD Dendritic Cell 0.154598454 0.001878822

TH COAD Purity 0.10713215 0.03070423

TH COAD B Cell -0.200050613 5.13E-05

TH COAD CD8+ T Cell -0.25658647 1.59E-07

TH COAD CD4+ T Cell 0.088914044 0.074961781

TH COAD Macrophage 0.009468472 0.849521887

TH COAD Neutrophil -0.177663614 0.000350112

TH COAD Dendritic Cell -0.177077027 0.000360321
