# Supplementary material for: Patient stratification based on urea cycle metabolism for exploration of combination immunotherapy in colon cancer
Source: BMC Cancer. 2022 Aug 13;22:883. doi: 10.1186/s12885-022-09958-7 (PMC9375340; doi:10.1186/s12885-022-09958-7)
Supplement: Supplementary file 2 — Additional file 2: Appendix D2. The immune infiltrates inclusters in the TCGA cohort. [file 12885_2022_9958_MOESM2_ESM.docx]

## Supplementary Materials

**Additional file 2: Appendix D2**

Appendix D2: The immune infiltrates in clusters in the TCGA cohort.

immune cor pvalue

T cell CD8+_TIMER -0.0977413393768593 0.0442732597560337

Neutrophil_TIMER -0.314064349392177 3.68981869187444e-11

Myeloid dendritic cell_TIMER -0.219435326908483 5.09551017537938e-06

B cell plasma_CIBERSORT 0.108312400550226 0.0257295425033899

T cell CD4+ memory activated_CIBERSORT -0.150670065244281 0.00186387926442384

T cell follicular helper_CIBERSORT -0.10806311947995 0.0260740935034683

Macrophage M1_CIBERSORT -0.113818495524471 0.0190592279320287

Myeloid dendritic cell activated_CIBERSORT -0.127076540582867 0.00880408961436678

Mast cell resting_CIBERSORT -0.185725213671961 0.000119795848305536

Neutrophil_CIBERSORT -0.306284299053063 1.16619602145926e-10

T cell CD8+_CIBERSORT-ABS -0.143933962866976 0.00297301521923883

T cell CD4+ memory resting_CIBERSORT-ABS -0.174015655648859 0.000318060554239389

T cell CD4+ memory activated_CIBERSORT-ABS -0.180915603137877 0.000180226794145177

T cell follicular helper_CIBERSORT-ABS -0.232120998897508 1.35369798793729e-06

T cell regulatory (Tregs)_CIBERSORT-ABS -0.105061694416243 0.0305446114019397

NK cell resting_CIBERSORT-ABS -0.12638421914899 0.00918255236403308

Macrophage M0_CIBERSORT-ABS -0.100195383721093 0.0391838821263602

Macrophage M1_CIBERSORT-ABS -0.191785543860548 7.05564847944325e-05

Macrophage M2_CIBERSORT-ABS -0.147340776057042 0.00235330255312491

Myeloid dendritic cell activated_CIBERSORT-ABS -0.134450421080906 0.0055553834269648

Mast cell resting_CIBERSORT-ABS -0.380159375407046 5.00440468720246e-16

Neutrophil_CIBERSORT-ABS -0.315500348171328 2.97271732465687e-11

Macrophage M1_QUANTISEQ -0.322810245724975 9.71745057553755e-12

Macrophage M2_QUANTISEQ 0.164990931675339 0.000647855900378654

Neutrophil_QUANTISEQ -0.104399212981594 0.0316152772117955

NK cell_QUANTISEQ 0.164871964652065 0.000653805550303772

T cell CD8+_QUANTISEQ -0.154274083906481 0.00144047071222537

T cell regulatory (Tregs)_QUANTISEQ -0.149311066154478 0.0020511486887091

uncharacterized cell_QUANTISEQ 0.187209456637183 0.000105389566539607

T cell_MCPCOUNTER -0.151753120152868 0.00172598879209854

T cell CD8+_MCPCOUNTER -0.107078136182631 0.0274746701666025

cytotoxicity score_MCPCOUNTER -0.296887724161998 4.4773855570751e-10

NK cell_MCPCOUNTER -0.1748391347971 0.000297541385360449

B cell_MCPCOUNTER -0.126263953813588 0.00924975159986933

Monocyte_MCPCOUNTER -0.178818123669229 0.00021467219832607

Macrophage/Monocyte_MCPCOUNTER -0.178818123669229 0.00021467219832607

Myeloid dendritic cell_MCPCOUNTER -0.142455680808092 0.00328541970104539

Neutrophil_MCPCOUNTER -0.265764939914655 2.75211613355816e-08

Cancer associated fibroblast_MCPCOUNTER -0.104753776346437 0.0310383290202066

Myeloid dendritic cell activated_XCELL -0.234329017207068 1.06635577052506e-06

B cell_XCELL -0.105180133698902 0.0303565030921204

T cell CD4+ memory_XCELL -0.159159680167334 0.00100683371126959

T cell CD8+ naive_XCELL 0.102036340713905 0.0356990371261872

T cell CD8+ central memory_XCELL -0.198014946508166 4.02450512485805e-05

Class-switched memory B cell_XCELL -0.157823093781741 0.00111160946445053

Myeloid dendritic cell_XCELL -0.30300341481687 1.87566749852236e-10

Endothelial cell_XCELL -0.164717557726958 0.00066160309081851

Granulocyte-monocyte progenitor_XCELL -0.104737655409823 0.0310643643705694

Hematopoietic stem cell_XCELL 0.13142500160765 0.0067287010981628

Macrophage_XCELL -0.207780118290555 1.61065730743273e-05

Macrophage M1_XCELL -0.197843985778638 4.08795620712746e-05

Macrophage M2_XCELL -0.174687161355315 0.000301232723233753

Monocyte_XCELL -0.269810091941245 1.65812950869744e-08

Neutrophil_XCELL -0.322240096383651 1.06145733557271e-11

B cell plasma_XCELL -0.154964101865615 0.00137026389086914

T cell CD4+ Th2_XCELL -0.152286679380415 0.00166155316018948

T cell regulatory (Tregs)_XCELL -0.173024398648162 0.000344509834290503

immune score_XCELL -0.198122129088467 3.98520110118669e-05

stroma score_XCELL -0.134340415434459 0.00559459342043095

microenvironment score_XCELL -0.171214709289815 0.000398157056523368

Cancer associated fibroblast_EPIC -0.129100460732334 0.00777609062734408

T cell CD4+_EPIC -0.103729482181335 0.0327300969060438

Macrophage_EPIC -0.217583718225413 6.14380619686855e-06

NK cell_EPIC -0.179093895175218 0.00020981549278215

uncharacterized cell_EPIC 0.131464216497955 0.00671217151867016
