# Supplementary material for: Patient stratification based on urea cycle metabolism for exploration of combination immunotherapy in colon cancer
Source: BMC Cancer. 2022 Aug 13;22:883. doi: 10.1186/s12885-022-09958-7 (PMC9375340; doi:10.1186/s12885-022-09958-7)
Supplement: Supplementary file 3 — Additional file 3: Appendix D3. The correlation of nine UCmetabolism-related gene expression with TMB in diverse cancer types. [file 12885_2022_9958_MOESM3_ESM.docx]

## Supplementary Materials

**Additional file 3: Appendix D3**

Appendix D3: The correlation of nine UC metabolism-related gene expression with TMB in diverse cancer types.

ID CancerType cor pValue

ALDOB ACC 0.00238633 0.983347671

ALDOB BLCA -0.010027559 0.839971131

ALDOB BRCA -0.060243376 0.059399936

ALDOB CESC -0.038026392 0.521847594

ALDOB CHOL 0.142857201 0.405878372

ALDOB COAD -0.014641303 0.771469862

ALDOB DLBC -0.062744644 0.71218357

ALDOB ESCA 0.299723198 0.000117921

ALDOB GBM -0.167086693 0.041678402

ALDOB HNSC -0.009764786 0.828950612

ALDOB KICH 0.028253609 0.823214384

ALDOB KIRC 0.022375422 0.684586265

ALDOB KIRP 0.103255348 0.085714628

ALDOB LAML 0.225383648 0.020183122

ALDOB LGG -0.219674598 6.85E-07

ALDOB LIHC -0.010020768 0.849928977

ALDOB LUAD -0.368788177 1.19E-17

ALDOB LUSC -0.061832018 0.172656919

ALDOB MESO 0.115809283 0.309461185

ALDOB OV -0.089891946 0.139219561

ALDOB PAAD -0.140057296 0.086305645

ALDOB PCPG -0.0504474 0.504881704

ALDOB PRAD 0.067137586 0.141073298

ALDOB READ -0.203977213 0.018978628

ALDOB SARC 0.053581028 0.413591726

ALDOB SKCM -0.073842999 0.111783908

ALDOB STAD -0.122471122 0.018760073

ALDOB TGCT 0.003547163 0.966224514

ALDOB THCA -0.122683308 0.00700407

ALDOB THYM 0.044001323 0.637594132

ALDOB UCEC 0.057012149 0.192145028

ALDOB UCS -0.180609005 0.182842165

ALDOB UVM -0.090596403 0.424168035

CCNB1 ACC 0.456117731 2.40E-05

CCNB1 BLCA 0.202180631 3.89E-05

CCNB1 BRCA 0.397033958 2.37E-38

CCNB1 CESC 0.175722305 0.002864128

CCNB1 CHOL 0.364807568 0.028697891

CCNB1 COAD 0.22176814 8.40E-06

CCNB1 DLBC 0.412991939 0.011620259

CCNB1 ESCA -0.049717521 0.532405762

CCNB1 GBM 0.161921252 0.048507614

CCNB1 HNSC 0.139088062 0.001985996

CCNB1 KICH 0.467694388 8.56E-05

CCNB1 KIRC 0.098977094 0.071691625

CCNB1 KIRP -0.083248477 0.166303592

CCNB1 LAML 0.103199544 0.292469296

CCNB1 LGG 0.406279406 2.47E-21

CCNB1 LIHC 0.145502861 0.005745123

CCNB1 LUAD 0.362778952 4.31E-17

CCNB1 LUSC 0.265759023 2.47E-09

CCNB1 MESO 0.249767152 0.026425193

CCNB1 OV 0.034192293 0.574469719

CCNB1 PAAD 0.383639522 1.16E-06

CCNB1 PCPG 0.157728785 0.036018372

CCNB1 PRAD 0.406549551 1.31E-20

CCNB1 READ 0.117419445 0.17996624

CCNB1 SARC 0.394925288 3.41E-10

CCNB1 SKCM 0.203101172 1.02E-05

CCNB1 STAD 0.547414324 3.72E-30

CCNB1 TGCT 0.103261492 0.216466747

CCNB1 THCA 0.148333345 0.001089955

CCNB1 THYM -0.712960854 1.93E-19

CCNB1 UCEC 0.252866216 4.21E-09

CCNB1 UCS 0.296387515 0.0265525

CCNB1 UVM 0.070385622 0.53498778

CD36 ACC -0.220248505 0.051124707

CD36 BLCA -0.005573272 0.910640993

CD36 BRCA -0.149476612 2.60E-06

CD36 CESC -0.112338524 0.057761024

CD36 CHOL -0.096869806 0.574094795

CD36 COAD 0.022009831 0.662359066

CD36 DLBC -0.103129445 0.542224672

CD36 ESCA -0.1642943 0.037892941

CD36 GBM -0.12014595 0.144424967

CD36 HNSC -0.013807979 0.759976761

CD36 KICH -0.158997278 0.20584633

CD36 KIRC 0.101396165 0.064991522

CD36 KIRP -0.033432666 0.578841658

CD36 LAML -0.065417042 0.505258946

CD36 LGG 0.108535943 0.015078894

CD36 LIHC 0.025071827 0.635884294

CD36 LUAD -0.23356916 1.17E-07

CD36 LUSC -0.09513848 0.035636074

CD36 MESO -0.127422281 0.263109943

CD36 OV 0.089832557 0.139482028

CD36 PAAD -0.475185741 7.05E-10

CD36 PCPG 0.012608325 0.867715493

CD36 PRAD -0.066815909 0.142992361

CD36 READ 0.030660768 0.727091552

CD36 SARC -0.012069969 0.853974847

CD36 SKCM -0.168822338 0.00025524

CD36 STAD -0.366088547 4.10E-13

CD36 TGCT -0.059585305 0.476511248

CD36 THCA -0.095273719 0.036526787

CD36 THYM 0.123361802 0.185137039

CD36 UCEC -0.020694501 0.636148224

CD36 UCS 0.073652366 0.589565157

CD36 UVM -0.062002457 0.584815144

CDC25C ACC 0.473856928 1.03E-05

CDC25C BLCA 0.274469024 1.74E-08

CDC25C BRCA 0.382853262 1.45E-35

CDC25C CESC 0.094414314 0.111102048

CDC25C CHOL 0.380651965 0.022004506

CDC25C COAD 0.1095415 0.029293053

CDC25C DLBC 0.280227596 0.093128472

CDC25C ESCA 0.12933763 0.103094622

CDC25C GBM 0.096446971 0.241960559

CDC25C HNSC 0.095399204 0.034387253

CDC25C KICH 0.492611546 3.07E-05

CDC25C KIRC 0.192931566 0.000407168

CDC25C KIRP -0.012802431 0.831712219

CDC25C LAML 0.030000043 0.760156081

CDC25C LGG 0.398625852 1.57E-20

CDC25C LIHC 0.142548672 0.006823839

CDC25C LUAD 0.409360754 9.59E-22

CDC25C LUSC 0.259544609 5.93E-09

CDC25C MESO 0.294830683 0.008348239

CDC25C OV 0.128106969 0.034705929

CDC25C PAAD 0.454760392 4.47E-09

CDC25C PCPG 0.138670974 0.065661231

CDC25C PRAD 0.487261001 4.15E-30

CDC25C READ 0.1579691 0.07044465

CDC25C SARC 0.341717827 7.74E-08

CDC25C SKCM 0.152013016 0.001007965

CDC25C STAD 0.466574141 2.72E-21

CDC25C TGCT 0.206509191 0.012699

CDC25C THCA 0.08017749 0.078659398

CDC25C THYM -0.706341202 5.79E-19

CDC25C UCEC 0.131925457 0.002454985

CDC25C UCS 0.14904859 0.272921809

CDC25C UVM 0.034745395 0.759624774

CDKN2A ACC 0.421564649 0.000109194

CDKN2A BLCA 0.180598448 0.000245496

CDKN2A BRCA 0.223111164 1.61E-12

CDKN2A CESC 0.041852311 0.480812689

CDKN2A CHOL -0.07793382 0.651421257

CDKN2A COAD -0.102864983 0.04076163

CDKN2A DLBC -0.005452821 0.974872642

CDKN2A ESCA 0.033712927 0.672138189

CDKN2A GBM -0.010445186 0.899392022

CDKN2A HNSC -0.120296561 0.007557466

CDKN2A KICH 0.261553586 0.035327526

CDKN2A KIRC 0.20751634 0.000139866

CDKN2A KIRP 0.00359049 0.952477498

CDKN2A LAML -0.196942114 0.043025273

CDKN2A LGG 0.083084772 0.063132451

CDKN2A LIHC 0.27306791 1.47E-07

CDKN2A LUAD 0.185063485 2.96E-05

CDKN2A LUSC -0.037262428 0.411459338

CDKN2A MESO -0.129443504 0.25555011

CDKN2A OV -0.034077194 0.575759278

CDKN2A PAAD 0.002764988 0.973120791

CDKN2A PCPG 0.115971434 0.124254343

CDKN2A PRAD 0.171636359 0.000152759

CDKN2A READ 0.094797637 0.279599312

CDKN2A SARC 0.066381594 0.310915661

CDKN2A SKCM 0.209514314 5.20E-06

CDKN2A STAD 0.071544639 0.170827255

CDKN2A TGCT -0.106053568 0.204232582

CDKN2A THCA 0.209576225 3.47E-06

CDKN2A THYM -0.07584658 0.416350258

CDKN2A UCEC -0.357178852 3.05E-17

CDKN2A UCS 0.181498031 0.180660973

CDKN2A UVM -0.035687324 0.753313275

CLCNKB ACC -0.20459126 0.07050611

CLCNKB BLCA 0.006505126 0.895778985

CLCNKB BRCA -0.06692881 0.036181805

CLCNKB CESC -0.118342467 0.045541322

CLCNKB CHOL -0.093907033 0.585916115

CLCNKB COAD -0.104504135 0.037642454

CLCNKB DLBC -0.097431006 0.56618697

CLCNKB ESCA -0.154250153 0.051474724

CLCNKB GBM -0.066669935 0.41917357

CLCNKB HNSC 0.089114189 0.048205494

CLCNKB KICH -0.113210087 0.36923335

CLCNKB KIRC -0.1371308 0.012382769

CLCNKB KIRP 0.136957962 0.02236979

CLCNKB LAML -0.098763622 0.313818348

CLCNKB LGG 0.041813203 0.35031582

CLCNKB LIHC -0.041813228 0.429626992

CLCNKB LUAD -0.068824811 0.123178702

CLCNKB LUSC 0.023192405 0.60928676

CLCNKB MESO -0.282910338 0.011526392

CLCNKB OV -0.16431438 0.006608664

CLCNKB PAAD -0.280841577 0.000477753

CLCNKB PCPG -0.160884892 0.032417504

CLCNKB PRAD -0.295946345 3.37E-11

CLCNKB READ -0.15632894 0.073450328

CLCNKB SARC -0.158598314 0.014944322

CLCNKB SKCM -0.221095794 1.48E-06

CLCNKB STAD -0.303292402 2.87E-09

CLCNKB TGCT 0.15699861 0.059313358

CLCNKB THCA -0.153934052 0.000696375

CLCNKB THYM 0.325248515 0.000346067

CLCNKB UCEC -0.226519207 1.55E-07

CLCNKB UCS -0.012685714 0.926067406

CLCNKB UVM -0.102399424 0.366073596

CYP11A1 ACC -0.089292567 0.43388286

CYP11A1 BLCA -0.00960788 0.846584082

CYP11A1 BRCA -0.1485929 2.99E-06

CYP11A1 CESC -0.181284546 0.002084538

CYP11A1 CHOL -0.154193029 0.369241978

CYP11A1 COAD -0.130148597 0.009520536

CYP11A1 DLBC 0.123310414 0.467161177

CYP11A1 ESCA -0.082366074 0.30045917

CYP11A1 GBM -0.024941806 0.762700497

CYP11A1 HNSC -0.119166229 0.008146289

CYP11A1 KICH 0.287630189 0.020163904

CYP11A1 KIRC -0.119145519 0.029970221

CYP11A1 KIRP -0.060915169 0.311527219

CYP11A1 LAML 0.127470506 0.192866054

CYP11A1 LGG 0.089926169 0.044233646

CYP11A1 LIHC 0.043983929 0.406045788

CYP11A1 LUAD -0.172164725 0.000104174

CYP11A1 LUSC -0.059638989 0.188422322

CYP11A1 MESO 0.062645737 0.583368133

CYP11A1 OV -0.031207043 0.608347089

CYP11A1 PAAD -0.219147312 0.006861475

CYP11A1 PCPG -0.008004692 0.915786536

CYP11A1 PRAD -0.333713883 5.30E-14

CYP11A1 READ -0.061933022 0.480520204

CYP11A1 SARC -0.088892315 0.174426474

CYP11A1 SKCM -0.136524913 0.003178727

CYP11A1 STAD -0.373808832 1.20E-13

CYP11A1 TGCT -0.065388173 0.43456739

CYP11A1 THCA -0.079057278 0.082940987

CYP11A1 THYM -0.17029999 0.066397257

CYP11A1 UCEC -0.159480608 0.000243541

CYP11A1 UCS -0.163683322 0.228042344

CYP11A1 UVM 0.009466383 0.933581851

FABP4 ACC -0.057929375 0.612079865

FABP4 BLCA -0.239881449 9.47E-07

FABP4 BRCA -0.208050966 4.81E-11

FABP4 CESC 0.02659911 0.654195756

FABP4 CHOL -0.333247591 0.047022205

FABP4 COAD 0.002293015 0.963719745

FABP4 DLBC -0.239687055 0.152682687

FABP4 ESCA -0.187403908 0.017645708

FABP4 GBM -0.294617028 0.000265046

FABP4 HNSC -0.044293932 0.3268546

FABP4 KICH -0.016122867 0.898566211

FABP4 KIRC -0.003902785 0.943521758

FABP4 KIRP -0.005644919 0.925350429

FABP4 LAML 0.142060588 0.146323829

FABP4 LGG 0.051070575 0.253868468

FABP4 LIHC -0.056915463 0.282148599

FABP4 LUAD -0.191950191 1.46E-05

FABP4 LUSC -0.136738032 0.002468914

FABP4 MESO -0.048959865 0.668297514

FABP4 OV 0.022316749 0.714060748

FABP4 PAAD -0.479382038 4.75E-10

FABP4 PCPG -0.110103237 0.144595676

FABP4 PRAD -0.132198806 0.00364221

FABP4 READ 0.025485967 0.771759928

FABP4 SARC -0.106633629 0.102975045

FABP4 SKCM -0.066238619 0.15384458

FABP4 STAD -0.295529724 7.49E-09

FABP4 TGCT -0.116603621 0.162500723

FABP4 THCA -0.103936519 0.022481471

FABP4 THYM -0.131451653 0.157732422

FABP4 UCEC -0.016506288 0.705927788

FABP4 UCS 0.002461918 0.985632635

FABP4 UVM -0.201825182 0.072607187

HAMP ACC -0.08225583 0.471109416

HAMP BLCA 0.07524719 0.129163837

HAMP BRCA 0.044691077 0.162123591

HAMP CESC 0.156841472 0.007877417

HAMP CHOL -0.383357105 0.021003094

HAMP COAD 0.087213934 0.08303291

HAMP DLBC 0.253674727 0.129601839

HAMP ESCA -0.054023013 0.497466857

HAMP GBM -0.088345919 0.283983409

HAMP HNSC -0.060452162 0.180665858

HAMP KICH -0.080001795 0.526409313

HAMP KIRC 0.097790516 0.075178352

HAMP KIRP 0.038594972 0.521622653

HAMP LAML 0.055844062 0.569643478

HAMP LGG 0.104261214 0.019584346

HAMP LIHC -0.132734265 0.011824721

HAMP LUAD -0.009596827 0.82999822

HAMP LUSC -0.126035013 0.005300337

HAMP MESO -0.137808444 0.225844372

HAMP OV -0.037926731 0.533384965

HAMP PAAD -0.19101781 0.018801833

HAMP PCPG -0.068801617 0.362854476

HAMP PRAD -0.064344017 0.158411387

HAMP READ -0.031760072 0.717713686

HAMP SARC 0.08608461 0.188490446

HAMP SKCM 0.050995056 0.272464565

HAMP STAD -0.073269216 0.160720824

HAMP TGCT -0.110431827 0.18606355

HAMP THCA -0.253565879 1.65E-08

HAMP THYM 0.181046872 0.050763858

HAMP UCEC -0.138917057 0.001418367

HAMP UCS -0.17216326 0.20451514

HAMP UVM -0.103859414 0.359242634

LEP ACC 0.030096837 0.792317562

LEP BLCA 0.022556183 0.649632687

LEP BRCA -0.157248218 7.53E-07

LEP CESC 0.136275907 0.021149417

LEP CHOL -0.103253475 0.548998076

LEP COAD 0.088254781 0.079409885

LEP DLBC 0.230732755 0.169459491

LEP ESCA -0.063434755 0.425506262

LEP GBM -0.137496646 0.094489357

LEP HNSC 0.094720628 0.035694956

LEP KICH 0.137101892 0.27612924

LEP KIRC -0.103410244 0.059813392

LEP KIRP 0.15699913 0.0087368

LEP LAML 0.218151475 0.024671598

LEP LGG 0.065548575 0.142897236

LEP LIHC -0.123853592 0.018897238

LEP LUAD 0.179928007 4.94E-05

LEP LUSC -0.061124304 0.177633307

LEP MESO -0.147783799 0.19368863

LEP OV 0.103298928 0.089064122

LEP PAAD -0.255263633 0.00156024

LEP PCPG -0.096415942 0.201736923

LEP PRAD -0.033492063 0.463193732

LEP READ 0.01075633 0.902575143

LEP SARC 0.047756151 0.466246306

LEP SKCM -0.016823225 0.717484422

LEP STAD -0.075500763 0.148319674

LEP TGCT -0.044157011 0.597932446

LEP THCA -0.09990569 0.028294855

LEP THYM 0.05297312 0.570552225

LEP UCEC 0.001625653 0.970357727

LEP UCS 0.181536554 0.180566885

LEP UVM 0.02335678 0.837064714

MMP1 ACC 0.361683474 0.001056241

MMP1 BLCA -0.14127846 0.004245982

MMP1 BRCA 0.268667347 1.15E-17

MMP1 CESC -0.028340735 0.633161181

MMP1 CHOL 0.143372466 0.404169471

MMP1 COAD 0.144890066 0.003859494

MMP1 DLBC 0.12778568 0.449569312

MMP1 ESCA 0.140276422 0.076857484

MMP1 GBM -0.248738579 0.002221548

MMP1 HNSC -0.136334446 0.00244138

MMP1 KICH 0.375511572 0.002053528

MMP1 KIRC 0.03024266 0.582940408

MMP1 KIRP -0.132974289 0.026623844

MMP1 LAML 0.133652937 0.171982258

MMP1 LGG 0.096201451 0.031326796

MMP1 LIHC 0.054951797 0.299111079

MMP1 LUAD 0.032975044 0.460566796

MMP1 LUSC -0.093864527 0.038191289

MMP1 MESO 0.14517495 0.201760796

MMP1 OV 0.05563252 0.3607181

MMP1 PAAD -0.078266953 0.33945906

MMP1 PCPG 0.017822619 0.813856986

MMP1 PRAD 0.014021309 0.758807877

MMP1 READ 0.103381922 0.238145609

MMP1 SARC 0.183264509 0.004827567

MMP1 SKCM -0.016534579 0.722129679

MMP1 STAD 0.157355561 0.002468191

MMP1 TGCT 0.004831454 0.954007344

MMP1 THCA 0.076635069 0.092842935

MMP1 THYM 0.316603426 0.000505679

MMP1 UCEC 0.123167985 0.004710761

MMP1 UCS 0.379443038 0.0039246

MMP1 UVM -0.080804571 0.476136882

NAT2 ACC 0.276665063 0.013577875

NAT2 BLCA 0.020914031 0.673614371

NAT2 BRCA -0.069551462 0.029468389

NAT2 CESC -0.096931497 0.101853043

NAT2 CHOL -0.031044712 0.857358504

NAT2 COAD -0.188333472 0.000163425

NAT2 DLBC -0.166890268 0.323516376

NAT2 ESCA 0.344623008 8.10E-06

NAT2 GBM -0.028043454 0.734234704

NAT2 HNSC -0.015525999 0.731201241

NAT2 KICH 0.056226961 0.656411768

NAT2 KIRC -0.007156985 0.896632658

NAT2 KIRP 0.040871061 0.497347015

NAT2 LAML 0.130620837 0.182005967

NAT2 LGG 0.196234464 9.67E-06

NAT2 LIHC -0.012802855 0.808979

NAT2 LUAD 0.122097535 0.006110085

NAT2 LUSC -0.041093237 0.365023755

NAT2 MESO -0.063072187 0.580802669

NAT2 OV 0.091973183 0.130258408

NAT2 PAAD 0.082653204 0.312999983

NAT2 PCPG -0.007682481 0.919164596

NAT2 PRAD -0.204965305 5.72E-06

NAT2 READ -0.051343937 0.558768201

NAT2 SARC -0.113827127 0.081631665

NAT2 SKCM -0.074423844 0.108985629

NAT2 STAD 0.132962779 0.01066961

NAT2 TGCT 0.040210168 0.631086544

NAT2 THCA -0.009186123 0.840575557

NAT2 THYM 0.358877072 7.09E-05

NAT2 UCEC -0.047620782 0.276089335

NAT2 UCS 0.165459076 0.222966909

NAT2 UVM -0.056100368 0.621116031

NOS2 ACC -0.091739773 0.42133821

NOS2 BLCA 0.014893836 0.764227213

NOS2 BRCA 0.058188504 0.068636292

NOS2 CESC -0.025956147 0.662031914

NOS2 CHOL 0.274893838 0.104677694

NOS2 COAD 0.140578191 0.005069922

NOS2 DLBC -0.25865339 0.122053069

NOS2 ESCA 0.263521318 0.000760215

NOS2 GBM 0.167166524 0.041579533

NOS2 HNSC 0.00094381 0.983340209

NOS2 KICH -0.375463716 0.002056457

NOS2 KIRC -0.17802637 0.001123503

NOS2 KIRP -0.086430773 0.15063654

NOS2 LAML 0.230773292 0.017312412

NOS2 LGG 0.027710546 0.536037908

NOS2 LIHC -0.08702822 0.09969715

NOS2 LUAD 0.016517413 0.711717427

NOS2 LUSC -0.078477441 0.083300413

NOS2 MESO 0.157119647 0.166708882

NOS2 OV 0.035613782 0.558656563

NOS2 PAAD -0.108448214 0.185014386

NOS2 PCPG 0.057427203 0.447713274

NOS2 PRAD -0.164950722 0.000275885

NOS2 READ 0.019756901 0.822094019

NOS2 SARC -0.035932017 0.583645695

NOS2 SKCM -0.139090535 0.002648139

NOS2 STAD 0.091379676 0.080003316

NOS2 TGCT 0.147887418 0.0758712

NOS2 THCA -0.126995203 0.005235259

NOS2 THYM 0.334323035 0.000229582

NOS2 UCEC -0.022483239 0.607257035

NOS2 UCS -0.024790142 0.856088055

NOS2 UVM 0.134071777 0.235755414

TH ACC -0.009428613 0.934273452

TH BLCA -0.049307013 0.320465567

TH BRCA 0.1847292 5.69E-09

TH CESC -0.021437517 0.718103136

TH CHOL -0.005180729 0.976077015

TH COAD -0.375113657 1.12E-14

TH DLBC 0.006590405 0.969119754

TH ESCA 0.090385006 0.255682989

TH GBM -0.143721819 0.080349158

TH HNSC -0.119038833 0.008215155

TH KICH -0.054656037 0.665432931

TH KIRC -0.100885951 0.066360138

TH KIRP 0.043270819 0.472415386

TH LAML 0.116040543 0.236194868

TH LGG -0.022947947 0.608350779

TH LIHC -0.146341285 0.005468239

TH LUAD 0.062028396 0.164824062

TH LUSC -0.032741036 0.470533195

TH MESO 0.25863844 0.021363535

TH OV -0.064312607 0.290566154

TH PAAD -0.174921126 0.031698216

TH PCPG 0.028039588 0.711030657

TH PRAD -0.133357024 0.003353832

TH READ -0.183085308 0.035616503

TH SARC -0.109411359 0.094262719

TH SKCM 0.004273419 0.926774483

TH STAD -0.034883257 0.504706106

TH TGCT 0.11483008 0.169031186

TH THCA -0.020275614 0.65702032

TH THYM 0.078591931 0.399632187

TH UCEC -0.116653016 0.007458989

TH UCS -0.075156871 0.581964804

TH UVM 0.192078049 0.087842025
