# Supplementary material for: Patient stratification based on urea cycle metabolism for exploration of combination immunotherapy in colon cancer
Source: BMC Cancer. 2022 Aug 13;22:883. doi: 10.1186/s12885-022-09958-7 (PMC9375340; doi:10.1186/s12885-022-09958-7)
Supplement: Supplementary file 4 — Additional file 4: Appendix S1-S3. Figure S1. The association of intersection prognostic genes with immunity. (A) The correlation of 14 UC metabolism-related genes expression level with immune infiltration level in colon cancer. (B) Comparisons of immune infiltration level among samples with copy number alteration and the normal of 14 UC metabolism-related genes. Figure S2. The consensus clustering analysis of clusters, GSEA of the Cluster 1, and the t-SNE and 3D PCA of risk groups in the TCGA cohort. (A) The consensus matrix legend and the other consensus matrixes of clusters in the TCGA cohort. (B) The CDF plots and the item tracking plot of consensus clustering matrix in the external validation cohort. (C) The GSEA of cluster 1 colon cancer in the TCGA cohort. (D) (E) The t-SNE and 3D PCA of risk groups in the TCGA cohort. Figure S3. Immunohistochemistry of four genes in normal colon tissue and cancerous colon tissue, respectively. (A) The protein expression of upregulated genes (CDKN2A and CLCNKB) in normal colon tissue and cancerous colon tissue respectively. The URL of CDKN2A was https://www.proteinatlas.org/ENSG00000147889-CDKN2A/tissue/colon#img,https://www.proteinatlas.org/ENSG00000147889-CDKN2A/pathology/colorectal+cancer#img. The URL of CLCNKB was https://www.proteinatlas.org/ENSG00000184908-CLCNKB/tissue/colon#img and https://www.proteinatlas.org/ENSG00000184908-CLCNKB/pathology/colorectal+cancer#img. (B) The protein expression of downregulated genes (NAT2 and NOS2) in normal colon tissue and cancerous colon tissue respectively. The URL of NAT2 was https://www.proteinatlas.org/ENSG00000156006-NAT2/tissue/colon#img and https://www.proteinatlas.org/ENSG00000156006-NAT2/pathology/colorectal+cancer#img. The URL of NOS2 was https://www.proteinatlas.org/ENSG00000007171-NOS2/tissue/colon#img and https://www.proteinatlas.org/ENSG00000007171-NOS2/pathology/colorectal+cancer#img. [file 12885_2022_9958_MOESM4_ESM.docx]

## Supplementary Materials

**Additional file 4**

**Appendix S1**


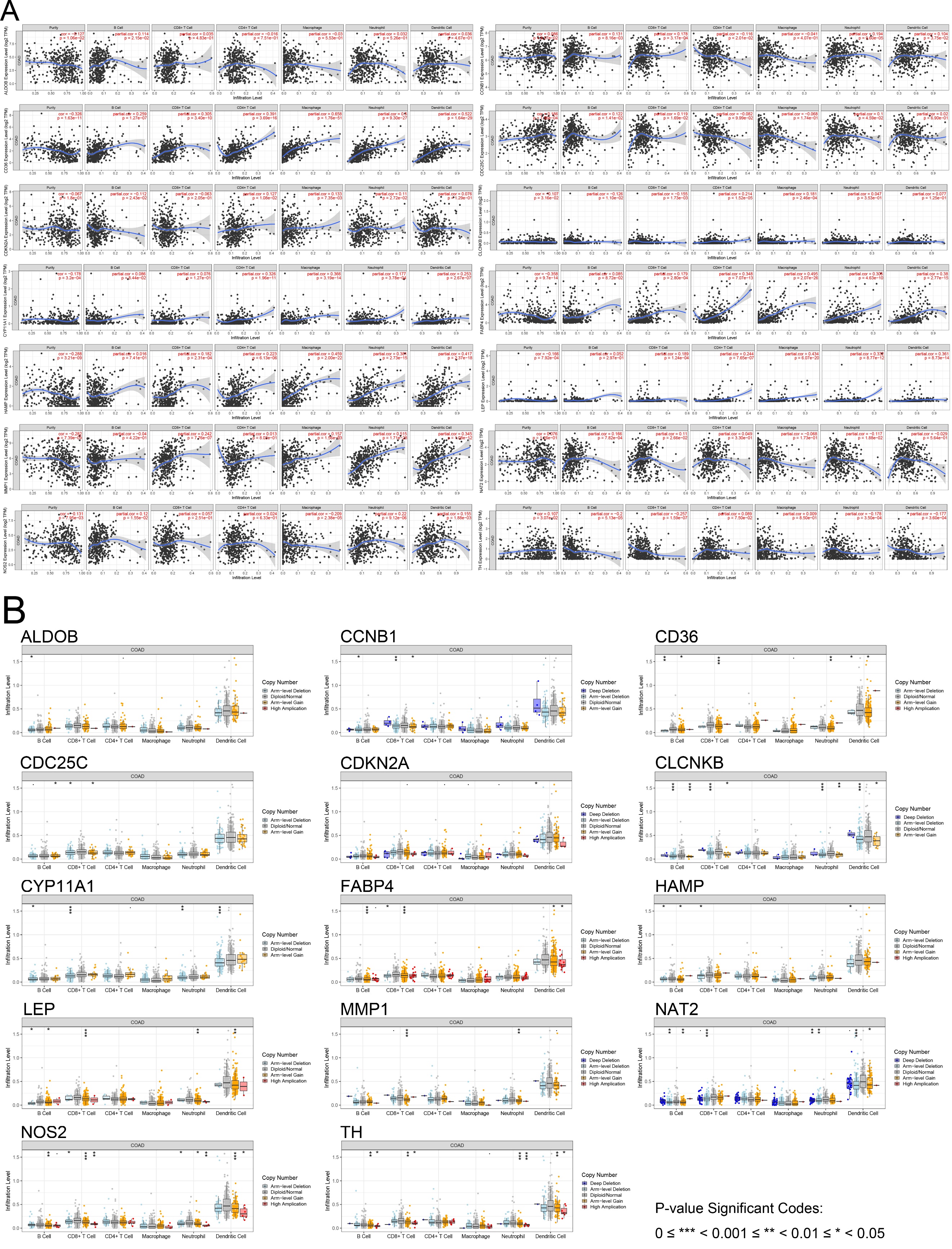


Figure S1: The association of intersection prognostic genes with immunity. (A) The correlation of 14 UC metabolism-related genes expression level with immune infiltration level in colon cancer. (B) Comparisons of immune infiltration level among samples with copy number alteration and the normal of 14 UC metabolism-related genes.

**Appendix S2**


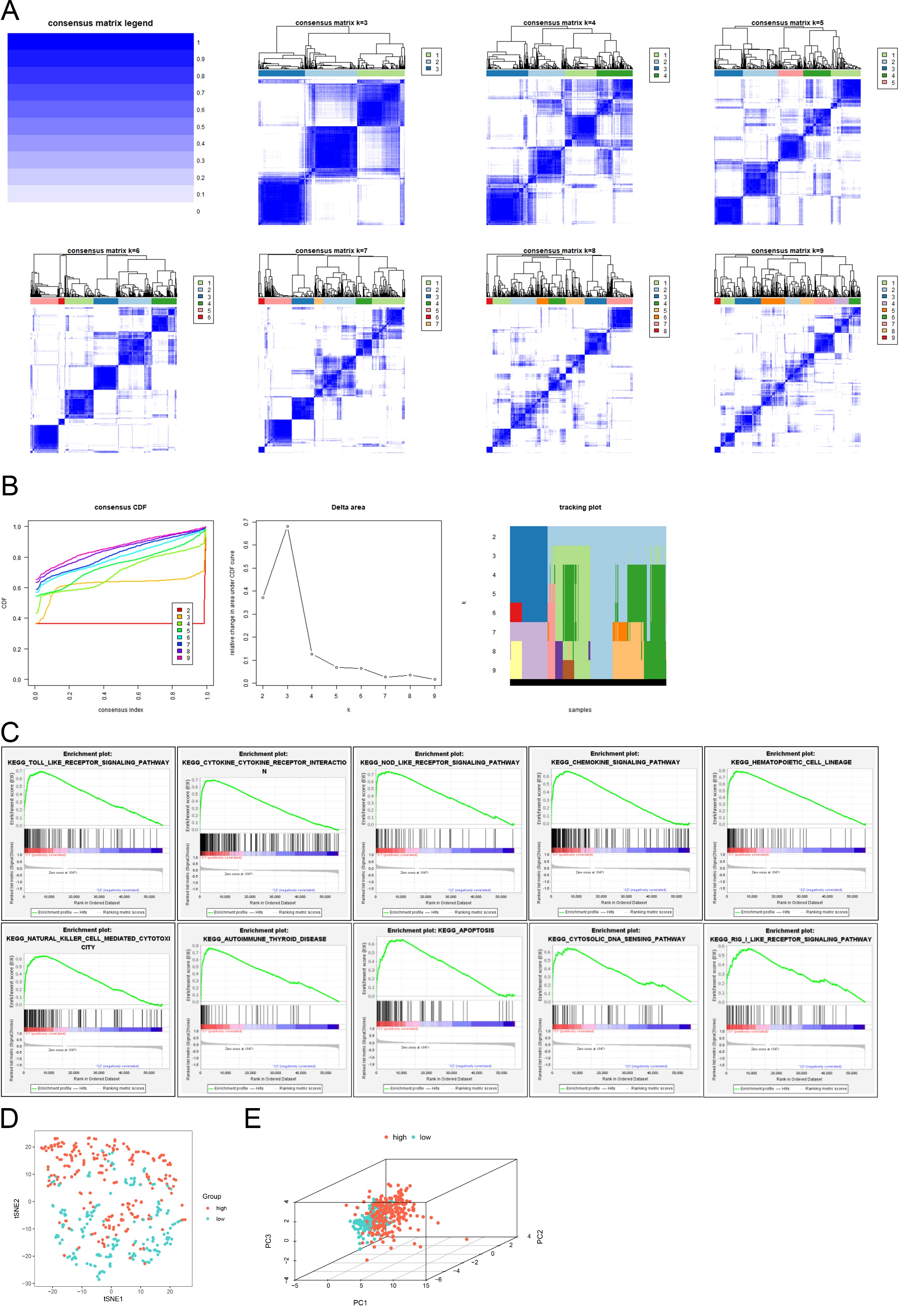


Figure S2: The consensus clustering analysis of clusters, GSEA of the Cluster 1, and the t-SNE and 3D PCA of risk groups in the TCGA cohort. (A) The consensus matrix legend and the other consensus matrixes of clusters in the TCGA cohort. (B) The CDF plots and the item tracking plot of consensus clustering matrix in the external validation cohort. (C) The GSEA of cluster 1 colon cancer in the TCGA cohort. (D)(E) The t-SNE and 3D PCA of risk groups in the TCGA cohort.

**Appendix S3**


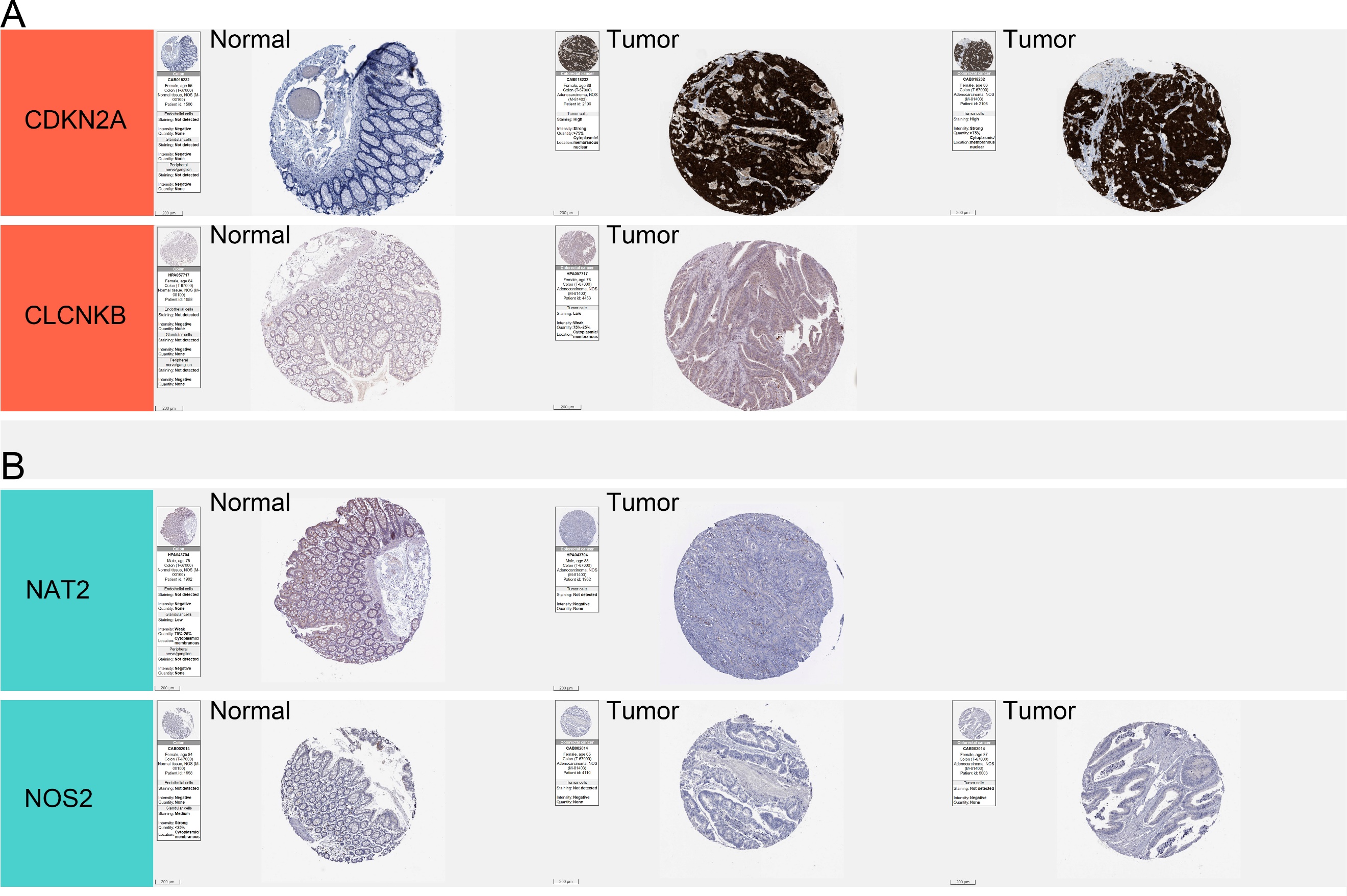


Figure S3: Immunohistochemistry of four genes in normal colon tissue and cancerous colon tissue, respectively. (A) The protein expression of upregulated genes (*CDKN2A* and *CLCNKB*) in normal colon tissue and cancerous colon tissue respectively. The URL of *CDKN2A* was https://www.proteinatlas.org/ENSG00000147889-CDKN2A/tissue/colon#img, https://www.proteinatlas.org/ENSG00000147889-CDKN2A/pathology/colorectal+cancer#img. The URL of *CLCNKB* was <https://www.proteinatlas.org/ENSG00000184908-CLCNKB/tissue/colon#img> and https://www.proteinatlas.org/ENSG00000184908-CLCNKB/pathology/colorectal+cancer#img. (B) The protein expression of downregulated genes (*NAT2* and *NOS2*) in normal colon tissue and cancerous colon tissue respectively. The URL of *NAT2* was <https://www.proteinatlas.org/ENSG00000156006-NAT2/tissue/colon#img> and <https://www.proteinatlas.org/ENSG00000156006-NAT2/pathology/colorectal+cancer#img>. The URL of *NOS2* was <https://www.proteinatlas.org/ENSG00000007171-NOS2/tissue/colon#img> and https://www.proteinatlas.org/ENSG00000007171-NOS2/pathology/colorectal+cancer#img.
