# Supplementary material for: Patient stratification based on urea cycle metabolism for exploration of combination immunotherapy in colon cancer
Source: BMC Cancer. 2022 Aug 13;22:883. doi: 10.1186/s12885-022-09958-7 (PMC9375340; doi:10.1186/s12885-022-09958-7)
Supplement: Supplementary file 5 — Additional file 5: Appendix T1. The urea cyclemetabolism-related genes. [file 12885_2022_9958_MOESM5_ESM.docx]

## Supplementary Materials

**Additional file 5: Appendix T1**

Appendix T1: The urea cycle metabolism-related genes.

| ACY1 |
| --- |
| ARG1 |
| ARG2 |
| ASL |
| ASS1 |
| BBOX1 |
| CKB |
| CKM |
| CKMT1B |
| CPS1 |
| DIO1 |
| GAMT |
| GATM |
| GLUD1 |
| OAT |
| ODC1 |
| OTC |
| PYCR1 |
| TTR |
| CEBPA |
| NAGS |
| NMRAL1 |
| SLC25A15 |
| SLC25A2 |
| AQP10 |
| AQP3 |
| AQP7 |
| AQP9 |
| SLC14A1 |
| SLC14A2 |
| UPK3A |
| ALDH18A1 |
| AZIN1 |
| AZIN2 |
| HNF4A |
| ADAMTS13 |
| C3 |
| CD46 |
| CFB |
| CFH |
| CFHR1 |
| CFHR3 |
| CFI |
| DBH |
| ELP1 |
| ERCC4 |
| ERCC6 |
| ERCC8 |
| FAN1 |
| THBD |
| PYCR3 |
| SARDH |
| SMS |
| SRM |
| GLS2 |
| SLC25A13 |
| F10 |
| F7 |
| GOT1 |
| GPT |
| ALDH4A1 |
| FH |
| GOT2 |
| LDHB |
| MDH1 |
| MDH2 |
| NOS1 |
| NOS2 |
| NOS3 |
| SLC25A12 |
| MMACHC |
| ALB |
| INS |
| TP53 |
| CYP2D6 |
| CYP2C19 |
| MMADHC |
| LEP |
| CYP3A4 |
| PPARG |
| CCND1 |
| PAH |
| HTR1A |
| APOE |
| SFTPC |
| PC |
| IL6 |
| ADIPOQ |
| TNF |
| CDK1 |
| MTHFR |
| HADHA |
| CYP2C9 |
| SFTPB |
| ADSL |
| CDKN1A |
| PRODH |
| CYP1A2 |
| CYP1A1 |
| MMAA |
| SLC7A7 |
| CDK2 |
| IGF1 |
| AKT1 |
| CYP2B6 |
| CDC25A |
| CYP2A6 |
| MMAB |
| CDC25C |
| CRP |
| ATM |
| LPL |
| CHEK2 |
| APOB |
| CDC20 |
| GHRL |
| BCKDHB |
| LMNA |
| ESR1 |
| CYP2C8 |
| CHEK1 |
| CDKN2A |
| CYP3A5 |
| CDK4 |
| CFTR |
| BRCA1 |
| APOA1 |
| INSR |
| CYP2E1 |
| FAH |
| G6PD |
| GLUL |
| GGT1 |
| GCK |
| CBS |
| CCNA2 |
| PCCA |
| PCCB |
| CDC6 |
| RETN |
| MYC |
| DPYD |
| ABCB11 |
| PPARA |
| RB1 |
| CDC27 |
| MMUT |
| SERPINA1 |
| CDC25B |
| CA5A |
| VDR |
| NAGLU |
| UGT1A1 |
| LIPC |
| MTOR |
| ACE |
| OGDH |
| PCK1 |
| CDC45 |
| CES1 |
| GALT |
| PGR |
| XDH |
| ACADM |
| SLC2A1 |
| HIF1A |
| BRCA2 |
| SERPINE1 |
| CCNB1 |
| PPARGC1A |
| LEPR |
| SIRT1 |
| PLK1 |
| LIPE |
| CDC14A |
| MIR122 |
| F2 |
| ABCC8 |
| DHFR |
| DLD |
| GNAS |
| ABCA1 |
| CDC37 |
| GCDH |
| EPHX2 |
| BGLAP |
| POMC |
| OPA3 |
| MED23 |
| CYP1B1 |
| HADH |
| ABCB1 |
| CDK6 |
| SOD1 |
| CETP |
| CRYAA |
| CAT |
| CD36 |
| CS |
| CLOCK |
| MDM2 |
| HMGCL |
| G6PC1 |
| PRL |
| MAPK1 |
| HPRT1 |
| ALDH2 |
| SLC2A4 |
| GH1 |
| VEGFA |
| HFE |
| LMBRD1 |
| AR |
| EPO |
| KCNJ11 |
| CCL2 |
| HSD17B10 |
| MEN1 |
| CYP19A1 |
| NAT2 |
| CYP7A1 |
| GCG |
| IL1B |
| LCAT |
| NR3C1 |
| ENPP1 |
| ETFDH |
| LDLR |
| SHBG |
| CASR |
| CYP27B1 |
| RAD51 |
| COMT |
| EGFR |
| PTGS2 |
| PTH |
| ACADVL |
| IRS1 |
| APOC3 |
| MCCC1 |
| SLC17A5 |
| POR |
| PON1 |
| ALDH5A1 |
| NBN |
| CCND3 |
| ADA |
| RBP4 |
| CDCA5 |
| TYR |
| CPT2 |
| TF |
| QDPR |
| TGFB1 |
| REN |
| PCNA |
| EGF |
| SLC12A1 |
| GSTM1 |
| LDHA |
| CLCNKB |
| FGF23 |
| TCF7L2 |
| IL10 |
| HAMP |
| GALE |
| STAT3 |
| NR1H2 |
| EP300 |
| SLC2A2 |
| PYGM |
| ACADS |
| AHCY |
| HSD11B1 |
| MPO |
| CTNNB1 |
| GNRH1 |
| AHR |
| PRKAA1 |
| HMGCR |
| TFRC |
| ADRB3 |
| TYMS |
| GK |
| MAT1A |
| HADHB |
| B2M |
| KCNJ1 |
| BCL2 |
| GSTP1 |
| CP |
| CASP3 |
| NR3C2 |
| SST |
| JAK2 |
| MAPK14 |
| GBA |
| AURKA |
| UQCRFS1 |
| PPARD |
| UCP2 |
| APP |
| NDUFS4 |
| GHR |
| IGF2 |
| CNR1 |
| FASN |
| FGFR1 |
| SLC34A1 |
| ATP7A |
| NQO1 |
| SLC12A3 |
| FANCD2 |
| HNF1A |
| DNMT1 |
| CYP11A1 |
| ARNTL |
| CDKN3 |
| ALOX5 |
| DGKE |
| PCSK9 |
| CAV1 |
| HSP90AA1 |
| APRT |
| NR1H4 |
| LPIN1 |
| HJV |
| ETFA |
| DLAT |
| EPHX1 |
| STAR |
| UGT1A9 |
| SLCO1B1 |
| AFP |
| CYP24A1 |
| PRKAR1A |
| CA2 |
| GSS |
| PRKAA2 |
| HIBCH |
| IGFBP3 |
| NRXN1 |
| HMOX1 |
| PPIG |
| GAPDH |
| RAD17 |
| CYP21A2 |
| NFKB1 |
| RPS27A |
| IFNG |
| DMGDH |
| MIR21 |
| SLC4A1 |
| APOA2 |
| AGXT |
| CYCS |
| RRM2B |
| ATR |
| DDC |
| HCRT |
| IVD |
| SLC25A20 |
| HK2 |
| GLS |
| FABP2 |
| MCCC2 |
| PARP1 |
| KRAS |
| ABL1 |
| RAD9A |
| H6PD |
| FANCA |
| SLC22A5 |
| ICAM1 |
| HBB |
| ABCC2 |
| CDKL5 |
| CDK5 |
| GNMT |
| SUOX |
| CTH |
| MCEE |
| PCK2 |
| HDAC1 |
| TKT |
| AMPD3 |
| MIR155 |
| IL2 |
| TAT |
| MCM5 |
| PIGA |
| DPP4 |
| IGFBP1 |
| CASP8 |
| PDP1 |
| CSNK1D |
| KL |
| HLCS |
| PNP |
| HP |
| MSH2 |
| TH |
| BCHE |
| ADRB2 |
| NDUFAF2 |
| CD4 |
| IGF1R |
| SP1 |
| AKR1A1 |
| H2AC18 |
| HSPA8 |
| ELANE |
| PLA2G6 |
| SPP1 |
| HRAS |
| CDKN2C |
| FLAD1 |
| TNFSF11 |
| VCP |
| NPY |
| GSTT1 |
| ALDOB |
| FTO |
| NR1I2 |
| CYP11B2 |
| BDNF |
| SCARB1 |
| CALCA |
| BAX |
| EHHADH |
| GSR |
| RAF1 |
| GSK3B |
| ALAD |
| ADK |
| SELE |
| HSPA1A |
| CSNK2A1 |
| ALDH9A1 |
| WRN |
| FLG |
| PIK3R1 |
| BRAF |
| BHMT |
| TPI1 |
| APC |
| H2AX |
| CXCL8 |
| PER1 |
| BCKDHA |
| RYR1 |
| FGF21 |
| RPA1 |
| ERBB2 |
| NPPA |
| STS |
| AKR1C3 |
| OPLAH |
| CYB5A |
| JUN |
| LBR |
| SLC40A1 |
| ACADSB |
| SOD2 |
| SRC |
| CSF3 |
| LPA |
| ECHS1 |
| WEE1 |
| ALDOA |
| SERPINA3 |
| TLR4 |
| FAS |
| FOXO1 |
| GAS1 |
| FANCM |
| TXN |
| TPO |
| KCNJ5 |
| SMC1A |
| MKI67 |
| ADH1B |
| CGA |
| KDR |
| NPM1 |
| CYP3A7 |
| GRHPR |
| AGT |
| SNCA |
| VWF |
| PRKCA |
| LIPG |
| DCXR |
| CYP2J2 |
| TYMP |
| PRPS1 |
| CYP27A1 |
| SLC6A8 |
| CDK9 |
| TNFRSF11B |
| OPRM1 |
| RAD50 |
| NR1I3 |
| MAPK10 |
| PIK3CG |
| CALR |
| IDH1 |
| KYNU |
| LEPQTL1 |
| SCN1A |
| HLA-B |
| TUBB |
| PIK3C2A |
| TP63 |
| TCN2 |
| PTPN1 |
| SLC22A12 |
| CDKN2D |
| GYS1 |
| CYP4A11 |
| PLA2G4A |
| MIR146A |
| SCNN1B |
| ANGPTL3 |
| VCAM1 |
| PDGFRB |
| MLH1 |
| CYP26A1 |
| MAPK8 |
| SHMT1 |
| EPRS1 |
| DNAH8 |
| FANCI |
| FLT3 |
| UBE3A |
| BCKDK |
| ATAD3A |
| NDUFAF5 |
| RET |
| AKR1B1 |
| PRKN |
| PTGS1 |
| TWNK |
| HDAC9 |
| MIR27A |
| CHKA |
| F5 |
| KCNJ10 |
| RPS6KB1 |
| ALDH1A1 |
| SCD |
| GLA |
| RHOA |
| DRD2 |
| SI |
| STK11 |
| UMPS |
| MMP1 |
| DBT |
| BMP6 |
| AHSG |
| MCM7 |
| MAP2K1 |
| FABP4 |
| H4-16 |
