# Supplementary material for: Patient stratification based on urea cycle metabolism for exploration of combination immunotherapy in colon cancer
Source: BMC Cancer. 2022 Aug 13;22:883. doi: 10.1186/s12885-022-09958-7 (PMC9375340; doi:10.1186/s12885-022-09958-7)
Supplement: Supplementary file 6 — Additional file 6: Appendix T2. The survival analysis ofprognostic genes on HPA for validation. [file 12885_2022_9958_MOESM6_ESM.docx]

## Supplementary Materials

**Additional file 6: Appendix T2**

Appendix T2: The survival analysis of prognostic genes on HPA for validation.

| ALDOB | Current cut off^i^ :9.83 | [Best expression cut offi :9.83](https://www.proteinatlas.org/ENSG00000136872-ALDOB/pathology/colorectal+cancer/COAD) | [Median expressioni :7.89](https://www.proteinatlas.org/ENSG00000136872-ALDOB/pathology/colorectal+cancer/COAD) | Median follow up time^i^ :1.87 | P score^i^0.012 | 5-year survival high^i^76% | 5-year survival low^i^54% |
| --- | --- | --- | --- | --- | --- | --- | --- |
| CCNB1 | Current cut off^i^ :47.3 | [Best expression cut offi :47.3](https://www.proteinatlas.org/ENSG00000134057-CCNB1/pathology/colorectal+cancer/COAD) | [Median expressioni :35.92](https://www.proteinatlas.org/ENSG00000134057-CCNB1/pathology/colorectal+cancer/COAD) | Median follow up time^i^ :1.87 | P score^i^0.00083 | 5-year survival high^i^80% | 5-year survival low^i^57% |
| CD36 | Current cut off^i^ :0.94 | [Best expression cut offi :0.94](https://www.proteinatlas.org/ENSG00000135218-CD36/pathology/colorectal+cancer/COAD) | [Median expressioni :0.46](https://www.proteinatlas.org/ENSG00000135218-CD36/pathology/colorectal+cancer/COAD) | Median follow up time^i^ :1.87 | P score^i^0.0020 | 5-year survival high^i^50% | 5-year survival low^i^67% |
| CDC25C | Current cut off^i^ :3.53 | [Best expression cut offi :3.53](https://www.proteinatlas.org/ENSG00000158402-CDC25C/pathology/colorectal+cancer/COAD) | [Median expressioni :3.27](https://www.proteinatlas.org/ENSG00000158402-CDC25C/pathology/colorectal+cancer/COAD) | Median follow up time^i^ :1.87 | P score^i^0.00044 | 5-year survival high^i^71% | 5-year survival low^i^57% |
| CDKN2A | Current cut off^i^ :1.15 | [Best expression cut offi :1.15](https://www.proteinatlas.org/ENSG00000147889-CDKN2A/pathology/colorectal+cancer/COAD) | [Median expressioni :1.07](https://www.proteinatlas.org/ENSG00000147889-CDKN2A/pathology/colorectal+cancer/COAD) | Median follow up time^i^ :1.87 | P score^i^0.0053 | 5-year survival high^i^60% | 5-year survival low^i^67% |
| CLCNKB | Current cut offi :0.02 | [Best expression cut offi :0.02](https://www.proteinatlas.org/ENSG00000184908-CLCNKB/pathology/colorectal+cancer/COAD) | [Median expressioni :0.02](https://www.proteinatlas.org/ENSG00000184908-CLCNKB/pathology/colorectal+cancer/COAD) | Median follow up time^i^ :1.87 | P score^i^0.036 | 5-year survival high^i^59% | 5-year survival low^i^69% |
| CYP11A1 | Current cut offi :0.07 | [Best expression cut offi :0.07](https://www.proteinatlas.org/ENSG00000140459-CYP11A1/pathology/colorectal+cancer/COAD) | [Median expressioni :0.03](https://www.proteinatlas.org/ENSG00000140459-CYP11A1/pathology/colorectal+cancer/COAD) | Median follow up time^i^ :1.87 | P score^i^0.012 | 5-year survival high^i^57% | 5-year survival low^i^65% |
| FABP4 | Current cut off^i^ :0.36 | [Best expression cut offi :0.36](https://www.proteinatlas.org/ENSG00000170323-FABP4/pathology/colorectal+cancer/COAD) | [Median expressioni :0.3](https://www.proteinatlas.org/ENSG00000170323-FABP4/pathology/colorectal+cancer/COAD) | Median follow up time^i^ :1.87 | P score^i^0.0023 | 5-year survival high^i^59% | 5-year survival low^i^68% |
| HAMP | Current cut off^i^ :0.11 | [Best expression cut offi :0.11](https://www.proteinatlas.org/ENSG00000105697-HAMP/pathology/colorectal+cancer/COAD) | [Median expressioni :0.08](https://www.proteinatlas.org/ENSG00000105697-HAMP/pathology/colorectal+cancer/COAD) | Median follow up time^i^ :1.87 | P score^i^0.0024 | 5-year survival high^i^52% | 5-year survival low^i^70% |
| LEP | Current cut off^i^ :0.02 | [Best expression cut offi :0.02](https://www.proteinatlas.org/ENSG00000174697-LEP/pathology/colorectal+cancer/COAD) | [Median expressioni :0.01](https://www.proteinatlas.org/ENSG00000174697-LEP/pathology/colorectal+cancer/COAD) | Median follow up time^i^ :1.87 | P score^i^0.000058 | 5-year survival high^i^51% | 5-year survival low^i^71% |
| MMP1 | Current cut off^i^ :7.69 | [Best expression cut offi :7.69](https://www.proteinatlas.org/ENSG00000196611-MMP1/pathology/colorectal+cancer/COAD) | [Median expressioni :28.31](https://www.proteinatlas.org/ENSG00000196611-MMP1/pathology/colorectal+cancer/COAD) | Median follow up time^i^ :1.87 | P score^i^0.014 | 5-year survival high^i^68% | 5-year survival low^i^48% |
| NAT2 | Current cut offi :3.19 | [Best expression cut offi :3.19](https://www.proteinatlas.org/ENSG00000156006-NAT2/pathology/colorectal+cancer/COAD) | [Median expressioni :3.02](https://www.proteinatlas.org/ENSG00000156006-NAT2/pathology/colorectal+cancer/COAD) | Median follow up time^i^ :1.87 | P score^i^0.00024 | 5-year survival high^i^73% | 5-year survival low^i^54% |
| NOS2 | Current cut off^i^ :3.92 | [Best expression cut offi :3.92](https://www.proteinatlas.org/ENSG00000007171-NOS2/pathology/colorectal+cancer/COAD) | [Median expressioni :7.19](https://www.proteinatlas.org/ENSG00000007171-NOS2/pathology/colorectal+cancer/COAD) | Median follow up time^i^ :1.87 | P score^i^0.022 | 5-year survival high^i^66% | 5-year survival low^i^58% |
| TH | Current cut off^i^ :0.63 | [Best expression cut offi :0.63](https://www.proteinatlas.org/ENSG00000180176-TH/pathology/colorectal+cancer/COAD) | [Median expressioni :0.22](https://www.proteinatlas.org/ENSG00000180176-TH/pathology/colorectal+cancer/COAD) | Median follow up time^i^ :1.87 | P score^i^0.045 | 5-year survival high^i^53% | 5-year survival low^i^67% |
| ADIPOQ | Current cut off^i^ :0 | [Best expression cut offi :0](https://www.proteinatlas.org/ENSG00000181092-ADIPOQ/pathology/colorectal+cancer/COAD) | [Median expressioni :N/A](https://www.proteinatlas.org/ENSG00000181092-ADIPOQ/pathology/colorectal+cancer/COAD) | Median follow up time^i^ :N/A | P score^i^N/A | 5-year survival high^i^N/A | 5-year survival low^i^N/A |
| IGF1 | Current cut off^i^ :0.09 | [Best expression cut offi :0.09](https://www.proteinatlas.org/ENSG00000017427-IGF1/pathology/colorectal+cancer/COAD) | [Median expressioni :0.05](https://www.proteinatlas.org/ENSG00000017427-IGF1/pathology/colorectal+cancer/COAD) | Median follow up time^i^ :1.87 | P score^i^0.26 | 5-year survival high^i^60% | 5-year survival low^i^65% |
| SCN1A | Current cut off^i^ : | [Best expression cut offi :0](https://www.proteinatlas.org/ENSG00000144285-SCN1A/pathology/colorectal+cancer/COAD) | [Median expressioni :N/A](https://www.proteinatlas.org/ENSG00000144285-SCN1A/pathology/colorectal+cancer/COAD) | Median follow up time^i^ :N/A | P score^i^N/A | 5-year survival high^i^N/A | 5-year survival low^i^N/A |
